# CNN-Based Identification of Parkinson’s Disease from Continuous Speech in Noisy Environments

**DOI:** 10.3390/bioengineering10050531

**Published:** 2023-04-26

**Authors:** Paul Faragó, Sebastian-Aurelian Ștefănigă, Claudia-Georgiana Cordoș, Laura-Ioana Mihăilă, Sorin Hintea, Ana-Sorina Peștean, Michel Beyer, Lăcrămioara Perju-Dumbravă, Robert Radu Ileșan

**Affiliations:** 1 Bases of Electronics Department, Faculty of Electronics, Telecommunications and Information Technology, Technical University of Cluj-Napoca, 400114 Cluj-Napoca, Romania; claudia.cordos@bel.utcluj.ro (C.-G.C.); laura.mihaila@bel.utcluj.ro (L.-I.M.); sorin.hintea@bel.utcluj.ro (S.H.); 2 Department of Computer Science, Faculty of Mathematics and Computer Science, West University of Timisoara, 300223 Timisoara, Romania; sebastian.stefaniga@e-uvt.ro; 3 Department of Neurology and Pediatric Neurology, Faculty of Medicine, University of Medicine and Pharmacy “Iuliu Hatieganu” Cluj-Napoca, 400012 Cluj-Napoca, Romania; popescu.ana.sorina@elearn.umfcluj.ro (A.-S.P.); lperjud@elearn.umfcluj.ro (L.P.-D.);; 4 Clinic of Oral and Cranio-Maxillofacial Surgery, University Hospital Basel, CH-4031 Basel, Switzerland; michel.beyer@usb.ch; 5 Medical Additive Manufacturing Research Group (Swiss MAM), Department of Biomedical Engineering, University of Basel, CH-4123 Allschwil, Switzerland

**Keywords:** speech assessment, hypokinetic dysarthria, artificial intelligence, Parkinson’s disease, continuous speech, noisy speech, pre-diagnosis, convolutional neural networks, spectrograms, Wiener filter

## Abstract

Parkinson’s disease is a progressive neurodegenerative disorder caused by dopaminergic neuron degeneration. Parkinsonian speech impairment is one of the earliest presentations of the disease and, along with tremor, is suitable for pre-diagnosis. It is defined by hypokinetic dysarthria and accounts for respiratory, phonatory, articulatory, and prosodic manifestations. The topic of this article targets artificial-intelligence-based identification of Parkinson’s disease from continuous speech recorded in a noisy environment. The novelty of this work is twofold. First, the proposed assessment workflow performed speech analysis on samples of continuous speech. Second, we analyzed and quantified Wiener filter applicability for speech denoising in the context of Parkinsonian speech identification. We argue that the Parkinsonian features of loudness, intonation, phonation, prosody, and articulation are contained in the speech, speech energy, and Mel spectrograms. Thus, the proposed workflow follows a feature-based speech assessment to determine the feature variation ranges, followed by speech classification using convolutional neural networks. We report the best classification accuracies of 96% on speech energy, 93% on speech, and 92% on Mel spectrograms. We conclude that the Wiener filter improves both feature-based analysis and convolutional-neural-network-based classification performances.

## 1. Introduction

Parkinson’s disease (PD) is a progressive neurodegenerative disorder (pathology where cells of the brain stop working or die) caused by dopaminergic neuron degeneration in the pars compacta of the substantia nigra from the ventral midbrain [1,2]. Furthermore, the presence, in the substantia nigra, of Lewy bodies containing alpha-synuclein is a clear neuropathological expression of PD [2].

The clinical presentation of patients with PD accounts, among others, for motor symptoms (e.g., tremor, bradykinesia, and rigidity), which could be seen as the last part of the cascade mechanism that starts with the upper-mentioned loss of dopaminergic neurons (substantia nigra), inducing reduced facilitation of voluntary movements and advancing to severe motor and non-motor symptoms. The last, non-motor symptoms (e.g., pain, fatigue, low blood pressure, restless legs, bladder and bowel problems, skin and sweating, sleep, eating, swallowing and saliva control, eye problems, foot care, dental health, mental health issues, mild memory and thinking problems, anxiety, dementia, depression, hallucinations and delusions, and speech and communication issues), have been gaining more and more attention in the last decades [3]. As we can comprehend, PD has a high diversity in clinical appearance, and new studies show that some of them (e.g., anxiety, depression, and anhedonia) could be related to serotonergic neurotransmission (non-dopaminergic systems) affecting up to 50% of the patients, with a clear impact on the quality of life [4,5,6,7,8]. 

The global incidence of PD increased from 2.5 million in 1990 to 6.1 million in 2016 [9], accounting for a 21.7% increase in the age-standardized rate of prevalence [10,11]. One million people have PD in the US alone, and the number is expected to reach 1.2 million by 2030 [12]. 

Based on the previously analyzed literature, we can argue that PD is highly challenging to diagnose and treat due to its myriad of clinical appearances. In this study, we focused on one of them, speech impairment, with the aim of supporting research in this field and clinicians in their quest for precision medicine. 

Parkinsonian speech impairment is defined by hypokinetic dysarthria, a motor disorder which affects the magnitude and velocity of the articulatory movements and the inter-articulator timing disturbances during speech production [13,14]. Hypokinetic dysarthria accounts for respiratory, phonatory, articulatory, and prosodic manifestations [15]. As such, Parkinsonian speech is characterized by voice blocking, reduced voice intensity, mono-pitch/mono-loudness oration, tremor phonation (changes in the energy and fundamental frequency), breathy/hoarse voice, and hypotonic phonation, as well as reduced stress and incorrect articulation [13,16,17,18,19,20]. Speaking tasks reported in the literature for the assessment of Parkinsonian speech are classified into sustained vowel phonation, diadochokinetic task (repetition of fast syllables, usually with occlusive consonants), and continuous speech (reading and/or monologue/free speech) [21,22]. We extend this classification with the addition of two further speech tasks, as identified in the literature: isolated words and short sentences. 

There is a prevalence of up to 89% in patients with PD who experience, among others, eloquent-speech difficulties, such as dysarthria (difficulty speaking due to brain damage, neuromuscular speech disorder) [23]. Unfortunately, clinical diagnosis for PD often materializes long after substantial neurophysiological damage has occurred as symptoms intensify over time. Altered speech is directly correlated with disability and poor outcomes resulting in reduced quality of life [7,8]. As speech impairment could be one of the first signs of PD [24]; timely identification is paramount for early intervention.

### 1.1. Related Work—Features Extraction

Feature classes for the objective assessment of hypokinetic phonation and articulatory impairment in PD are presented in Table 1, categorized by the speaking task. 

Voice blocking is assessed using phonetic and phonologic speech features: pause count, pause duration, speech rate, etc. [25,26], from continuous speech. 

Reduced speech loudness/intensity and mono-pitch and mono-loudness oration are assessed from prosody [27] based on pitch, i.e., fundamental frequency (*f*_0_) and speech intensity (*I*)/energy (*E*), respectively [28], taken in standard deviation. 

Tremor phonation (and voice quality) is assessed on sustained vowels [13], isolated words, or short sentences [29,30,31], in terms of speech prosody: intensity/energy variation, fundamental frequency variation, and harmonic-to-noise ratio (HNR) [32]. 

Articulatory impairment is assessed by means of formant analysis, usually on sustained vowel phonation [13,33,34] and isolated words [31].

As illustrated, most of the literature references handle sustained vowel phonation and diadochokinetic speech tasks, along with isolated word and short sentence utterings. There are very few references to Parkinsonian speech assessment and identification in continuous speech. 

Khan et al. argue in [35] that the assessment and identification of PD on continuous speech leads to better results by using Mel-frequency cepstral coefficients (MFCCs). Indeed, MFCC was employed, in addition to prosody, noise, formant, and cepstral analysis, for running speech assessment by Orzoco et al. in [36]. As for another example, Laganas et al. also employed MFCC besides pitch, pitch onset, and pitch offset for running speech assessment in PD [28]. 

Further on, Parkinsonian speech can be assessed using time-domain features, e.g., (short-term) energy and zero crossing rate, to evaluate voice activity [37]. On the other hand, Parkinsonian speech can be assessed using frequency-domain features, e.g., skewness and kurtosis [37], as well as MFCCs and the derivatives of MFCCs to evaluate spectrum shape [38].

The features reported in the literature for Parkinsonian speech assessment are listed in Table 2, categorized by the feature classes. 

### 1.2. Related Work—Classifiers

Regarding Parkinsonian speech identification, several classifiers have been reported in the literature: Multilayer Perceptron (MLP), Extreme Gradient Boosting (XGBoost), K-Nearest Neighbor (KNN), and Random Forest (RF) [39], support vector machines (SVMs), artificial neural networks (ANNs)/convolutional neural networks (CNNs) [40]. SVMs and CNNs exhibit the most widespread employment: SVMs are preferred for vowel and syllable classification, whereas CNNs are preferred for sequences of text. 

For exemplification, an SVM model with a hybrid CS-PSO parameter optimization method was used by Kaya in [41] and achieved a 97.4% accuracy on the classification of voice measurements. 

An SVM was also employed by Yaman et al. in [42], along with k-NN, for the automatic detection of PD from vowels. In this study, a statistical pooling method was applied to increase the size of the dataset. Then, the reported accuracy accounted for 91.25% in the case of SVM and 91.23% in the case of KNN.

Appakaya et al. employed the fine Gaussian SVM in [43] for the classification of Mel-frequency cepstral coefficients (MFCCs) extracted from three isolated words clustered into nine groups depending on the vowel content and achieved accuracy values that were between 60% and 90%. The study analyzed both fixed-width and pitch synchronous speech segmentation. 

Hoq et al. proposed two hybrid models which integrate the Principal Component Analysis (PCA) and the deep neural network (DNN) of a Sparse Autoencoder (SAE) into an SVM in [39] and achieved an accuracy of 89.4% and 94.4%, respectively, for the detection of Parkinsonian speech based on the patient’s vocal features. 

As an alternative to SVMs, which perform Parkinsonian speech identification based on features sets, CNNs perform Parkinsonian speech identification by solving an image classification problem. 

For exemplification, Suhas et al. employed CNNs to perform spectrogram-based classification of dysarthria into three classes, amyotrophic lateral sclerosis (ALS), Parkinson’s disease (PD), and healthy controls (HC), and reported accuracy values above 80% [44].

Vaiciukynas et al. employed CNNs for Parkinsonian speech detection from a four-word sentence, achieving the best accuracy, i.e., 85.9% (equal error rate of 14.1%) [38]. In their work, the CNN was applied to classify the spectrograms of nine feature maps, including speech spectrograms; Mel frequency spectral coefficients—with the first and second derivative; Mel frequency cepstral coefficients; and linear predictive coding coefficients. 

Gómez-Vilda et al. proposed a Random Least Squares Feed-Forward Network (RLSFN), namely an ANN classifier with stochastic and least-square learning methods for weight adaptation, in [13] for PD detection from sustained vowel recordings, with an accuracy over 99.4%. PD detection was performed based on the speech articulation neuro-mechanics, i.e., absolute kinematic velocity of the jaw-tongue system assessed in [13] by signal energy and formants. 

### 1.3. Present Study

The topic of this article targets AI-based speech assessment for the identification of Parkinsonian speech. In previous work, we considered speech assessment in the framework of a decision support system for PD pre-diagnosis [45]. In the present study, we went further and focused on parkinsonian speech classification from running speech with the aim to facilitate the development of decision support systems for pre-diagnosis in neuroscience.

The literature review shows an abundance of reports on PD identification from short speech segments, i.e., vowels, syllables, and short words/sentences, mostly recorded in a laboratory environment. On the other hand, sample recordings in ambient conditions and PD identification from continuous speech are pursued less in the literature. Moreover, none of the reviewed solutions attempts to solve this problem by using CNN [46,47,48]. As such, the speech assessment workflow proposed in this article is aimed towards the assessment of continuous speech acquired in a noisy environment.

Our work is based on the premises that PD is identifiable from speech through loudness, intonation, phonation, prosody, and articulation. For this purpose, in our study, we performed an extensive investigation into phonological features, prosody features, time-domain features, frequency-domain features, and LPC analysis for formant extraction. Furthermore, we argue that the Parkinsonian traits identified with the feature-based speech analysis are contained in the speech, speech energy, and Mel spectrograms. Thus, we consider the spectrograms to be excellent candidates for CNN-based classification.

The novelty of this work is twofold. First, speech assessment was performed on samples of continuous speech, rather than utterings of sustained vowels, syllables, isolated words, or short sentences, as previously reported in the literature.

Second, we recorded the speech samples in a clinic, in the examination room—an inherently noisy environment, with no prior measures taken for soundproofing and noise reduction. On the one hand, this allowed us to investigate the presence of Parkinsonian speech attributes in the noisy signal. On the other hand, we were able to analyze and quantify the applicability of an optimal filter—the Wiener filter [49,50,51], in our work—for speech denoising in the context of Parkinsonian speech identification.

It should be noted that the speech samples used for the Parkinsonian speech assessment and CNN training were recorded from Romanian speaking patients and healthy controls (HCs) from our targeted study group. The dataset was constructed following a research protocol we devised ourselves, in contrast to publicly available third-party speech databases where we have no control over the acquisition and processing protocol.

## 2. Materials and Methods

Our methodology for AI-based Parkinsonian speech identification follows speech acquisition, speech processing, an investigation on feature extraction and feature assessment, and finally CNN-based spectrogram classification.

### 2.1. Speech Acquisition Protocol

**Figure 1:**
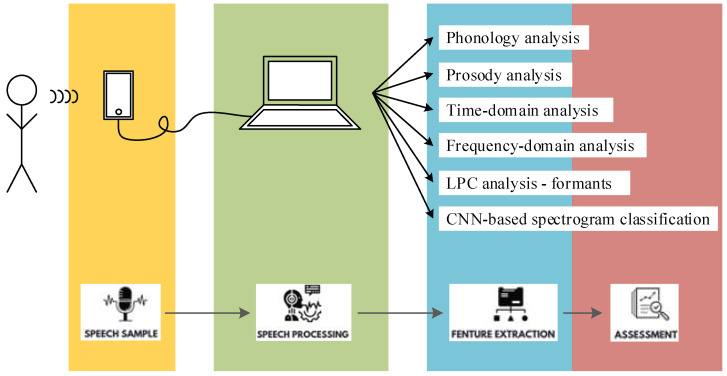
Speech acquisition and assessment protocol in the study of AI-based Parkinsonian speech identification.

The protocol adopted for speech acquisition and assessment is depicted in the workflow in Figure 1. 

Speech acquisition was performed indoors, in a clinical environment, in the examination room of the Neurology Department. No special measures were taken for soundproofing or noise reduction in the examination room. 

The study group consisted of twenty-seven subjects: sixteen PD patients and eleven healthy controls (HCs). The PD group included ten males and six females. The HC group included six males and five females. The healthy controls did not have any previously diagnosed neurodegenerative disorder or logopedic condition. 

The subjects were provided with an A4 printout with the date of evaluation and a 31-word text sequence in the Romanian language that they were asked to read out. The evaluator recorded the subjects’ speaking with a 44.1 kHz sampling frequency, using the sound recorder from an Android smartphone device, and downloaded the recording onto a laptop for speech processing and assessment. 

Speech assessment was performed in this study in terms of phonology, prosody, time-domain, frequency-domain, and LPC analyses for formant extraction, as well as CNN-based classification of the speech, speech energy, and Mel spectrograms.

### 2.2. Proposed Workflow for Speech Processing and Assessment 

**Figure 2:**
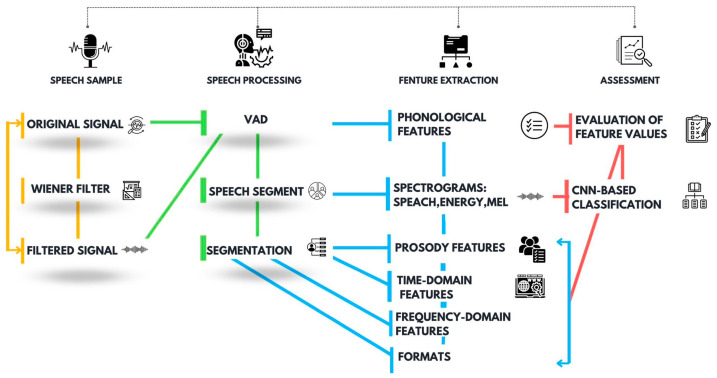
Proposed speech processing and assessment workflow, aiming for the identification of Parkinsonian speech following feature-based assessment and CNN-based classification.

Speech processing and assessment was performed in the MATLAB environment following the block diagram from Figure 2, which accounts for speech sample importation, speech processing, feature extraction, and assessment. 

Considering that the speech acquisition was performed in the clinic, which is an inherently noisy environment, a noise suppression stage implemented in this work with the Wiener filter was envisioned in the speech processing and assessment workflow. To investigate the effects of noise suppression on the speech assessment outcome, the same assessment procedure was applied to both original and filtered signals for comparison.

As indicated in Figure 2, a voice activity detector (VAD) was employed to discriminate speech from silence and pauses and, thus, to identify the speech segments. An energy-based VAD implementation was considered in this work. The VAD implementation assumes speech signal segmentation with 20 ms non-overlapping rectangular windows and the extraction of the signal energy (*enrg*) in each segment. The energy comparison threshold was set empirically to 1/10 of the maximum signal energy. Accordingly, speech activity is characterized by a larger signal energy in contrast to silence [52]. The evaluation of the Parkinsonian speech attributes is then performed on the extracted speech segments.

The Parkinsonian speech assessment features targeted in this work are listed in Table 3. The phonological feature extraction procedure is straightforward, following voice activity detection, and relies basically on counting the utterings and pauses. Prosody, time domain, frequency domain, formant analyses, and spectrogram classification, on the other hand, only target the active segments of speech. For this purpose, we considered extracting the segments of speech from the speech samples. 

For each of the extracted speech segments, we generated the speech spectrogram, speech energy spectrogram, and Mel spectrogram. The spectrograms were then applied for CNN-based classification. 

Finally, feature extraction was performed on each of the extracted speech segments. For this purpose, we considered segmentation with 20 ms rectangular windows and 50% overlap [37], followed by specific prosody, time-domain, frequency domain, and formant extraction techniques. 

#### 2.2.1. Mathematical Formula of the Wiener Filter

Adaptive linear filtering is based on the theory of minimum least square error filters and is applied in a variety of domains, e.g., linear prediction, echo cancellation, system identification, channel equalization, etc. 

In adaptive filters, the aim of parameter adaptation is to minimize the estimation error, *e*(*t*), between the desired signal, *s*(*t*), and the filtered signal, *ŝ*(*t*):

(1) $$e\left(t\right)=s\left(t\right)-\hat{s}\left(t\right).$$

**Figure 3:**
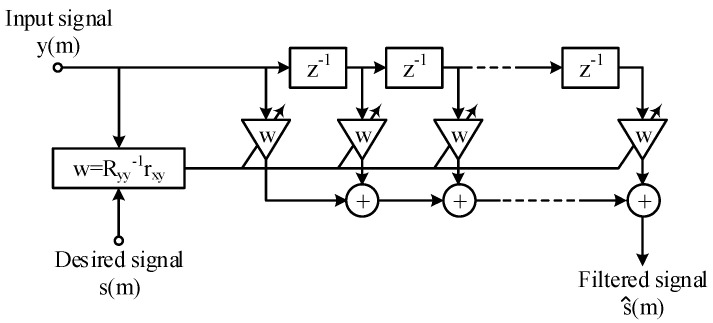
Block diagram of the Wiener filter implemented on the FIR filter topology.

In this paper, the Wiener filter is implemented on the FIR filter topology in Figure 3. Adaptivity assumes having the filter parameters recalculated in an automatic fashion to account for the statistical characteristics of the input signal and noise during the filtering process [49,50,51]. 

Our choice for the FIR filter is motivated by the stability of the topology, as well as ease in computing the filter weights. 

##### Time-Domain Equations

The filter transfer function is given by the following convolution:

(2) $$\hat{s}\left(n\right)=\sum_{k=0}^{N-1}w_{k}\cdot y\left(n-k\right),$$

Alternatively, it is expressed using vector notation:

(3) $$\hat{s}\left(n\right)=w^{t}\cdot y,$$

where *w* = [*w_i_*], *i* = 0…*N* − 1 is the coefficient vector, and *y* is the input vector to the FIR filter. The estimation error (1) is then expressed in discrete time as

(4) $$e\left(n\right)=s\left(n\right)-\hat{s}\left(n\right)=s\left(n\right)-w^{t}\cdot y.$$

The Wiener filter operates towards minimizing the mean square error (MSE); thus, we have the following:

(5) $$E\left[e^{2}\left(n\right)\right]=E\left[{\left(s\left(n\right)-w^{T}y\right)}^{2}\right]=E\left[s^{2}\left(n\right)\right]-2w^{T}E\left[y\cdot s\left(n\right)\right]+w^{T}E\left[y\cdot y^{T}\right]w,$$

where *E*[.] is the expectation operator. Then, one can identify that

(6) $$r_{ss}\left(0\right)=E\left[s^{2}\left(n\right)\right]$$

is the variance of the desired signal under the assumption that the mean of *s* is 0. Under the additional assumption that the input signal, *y*, and the desired responses are jointly stationary [51], one will further identify that

(7) $$r_{ys}\left(n\right)=E\left[y\cdot s\left(n\right)\right]$$

is the cross-correlation vector between the input and the desired signals, and

(8) $$R_{yy}=E\left[y\cdot y^{t}\right]$$

is the input signal autocorrelation matrix. The MSE is then rewritten as follows:

(9) $$E\left[e^{2}\left(n\right)\right]=r_{ss}\left(0\right)-2w^{T}r_{ys}+w^{T}R_{yy}w.$$

Under the Wiener theory, the filter optimization criterion is the least mean square error (LMSE) [51]. The MSE given in (9) is a second-order function in *w*, which has a single minimum that is determined by

(10) $$\frac{\partial }{\partial w}E\left[e^{2}\left(n\right)\right]=-2r_{ys}+2w^{T}R_{yy}=0,$$

which resolves to the Wiener coefficient vector, *w*, which satisfies the LMSE criterion:

(11) $$w={R_{yy}}^{-1}r_{ys}$$

In the case of additive noise, *n*, namely

(12) y(n)=s(n)+n(n),

and assuming that the signal and noise are uncorrelated, we obtain the following:

(13) $$r_{sn}=0,$$

whereas the noisy and noise-free signal are correlated:

(14) $$r_{ss}=r_{sy},$$

Then, it follows that [49]

(15) $$R_{yy}=R_{ss}+R_{nn}.$$

Substituting (14) and (15) in (11) yields the following:

(16) $$w={\left(R_{ss}+R_{nn}\right)}^{-1}\cdot r_{ss}$$

which defines the optimal linear filter for additive noise suppression [49].

##### Frequency-Domain Equations

In the frequency domain, the Wiener filter output *Ŝ*(*f*) is expressed as follows:

(17) $$\hat{S}\left(f\right)=Y\left(f\right)\cdot W\left(f\right)$$

which defines the error signal *E*(*f*) as follows:

(18) $$E\left(f\right)=S\left(f\right)-\hat{S}\left(f\right)=S\left(f\right)-Y\left(f\right)\cdot W\left(f\right).$$

The MSE is then expressed as follows:

(19) $$E\left[{\left|E\left(f\right)\right|}^{2}\right]=E\left[\left(S\left(f\right)-Y\left(f\right)\cdot W\left(f\right)\right)*\left(S\left(f\right)-Y\left(f\right)\cdot W\left(f\right)\right)\right],$$

where *E*[.] is the expectation operator, and * is the complex-conjugated product. Then, one can identify the following:

(20) $$P_{YY}\left(f\right)=E\left[Y\left(f\right)\cdot Y^{*}\left(f\right)\right],$$

as the power spectrum of *Y*(*f*), and

(21) $$P_{SY}\left(f\right)=E\left[S\left(f\right)\cdot Y^{*}\left(f\right)\right],$$

as the cross-power spectrum of *Y*(*f*) and *S*(*f*) [49].

The derivation of the Wiener coefficients under the LMSE criterion requires us to equate the MSE derivative to 0:

(22) $$\frac{\partial E\left[{\left|E\left(f\right)\right|}^{2}\right]}{\partial W\left(f\right)}=-2\cdot P_{SY}\left(f\right)+2\cdot W\left(f\right)\cdot P_{YY}\left(f\right)=0.$$

The transfer function of the Wiener filter is then expressed as follows:

(23) $$W\left(f\right)=\frac{P_{sy}\left(f\right)}{P_{yy}\left(f\right)}.$$

In the case of additive noise, the filter input signal is expressed in the frequency domain:

(24) Y(f)=S(f)+N(f),

where *N*(*f*) is the noise spectrum. Under the assumption that the signal and noise are uncorrelated, whereas the noisy signal and noise-free signal are correlated, as were the assumptions for the time-domain analysis, the Wiener filter is rewritten as follows:

(25) $$W\left(f\right)=\frac{P_{ss}\left(f\right)}{P_{ss}\left(f\right)+P_{nn}\left(f\right)},$$

where *P_ss_*(*f*) and *P_nn_*(*f*) are the signal and noise power spectra, respectively [49]. Dividing both nominator and denominator by *P_nn_*(*f*) yields the following:

(26) $$W\left(f\right)=\frac{\zeta \left(f\right)}{\zeta \left(f\right)+1},$$

where *ζ*(*f*) is the signal-to-noise ratio defined in terms of power spectra [49,50]. The MATLAB implementation of the Wiener filter, empowered in our work, follows the mathematical formula derived by (26).

##### Wiener Filter Performance Metrics

An objective evaluation of the Wiener filter noise suppression performance was performed in this work by using the signal-to-noise ratio (SNR) and signal-to-noise ratio improvement (*SNRI*) as speech enhancement measures, and the mean square error (MSE) as signal fidelity measure [52,53,54]. Each is defined as follows. 

The SNR is estimated in dB according to the definition of the global SNR as the logarithm of the signal (*P_signal_*) and noise (*P_noise_*) power ratio:

(27) $$SNR\left[dB\right]=10\cdot \mathrm{lg}\left(\frac{P_{signal}}{P_{noise}}\right),$$

where the noise power, *P_noise_*, is determined from the silence segments and the signal power, *P_signal_*, is determined from the speech activity segments, as discriminated by the voice activity detector [52]. Note that, although *P_signal_* contains the power of both speech and noise, the SNR estimated with (34) is relevant to evaluate the noise suppression performances of the Wiener filter. Large SNR values imply that speech magnitude is considerably larger than noise, whereas small SNR values imply that the noise magnitude is rather large in comparison to speech magnitude. 

The SNR is expressed for both original and filtered signals. Then, we estimate the *SNRI* as follows:

(28) $$SNRI\left[dB\right]=SNR_{original}\left[dB\right]-SNR_{filtered}\left[dB\right],$$

indicating the improvement of the speech sample.

Finally, the MSE is computed according to the following:

(29) $$MSE=\frac{1}{n}\overset{n}{\underset{i=1}{\sum }}{\left(s_{i}-\hat{s_{i}}\right)}^{2}.$$

#### 2.2.2. Feature Extraction for Parkinsonian Speech Assessment

The feature extraction stages applied for phonological, prosody, time-domain, frequency-domain, and LPC analyses, sequentially, are described as follows. 

##### Phonological Analysis

A phonological analysis of the speech signal, aiming for the identification of Parkinsonian speech phonology, was performed in in this work in terms of the number of utterings (*n_utterings_*), number of pauses (*n_pauses_*), speech rate (*r_speech_*), and pause duration (*t_pause_*). 

Phonological feature extraction is straightforward, following voice activity detection, and it is described as follows:

- The uttering count corresponds to the number of detected voice activities,
- The pause count corresponds to the number of detected pauses,
- The speech rate, expressed in words/minute, is determined as the number of utterings expressed throughout the complete speech duration,
- The pause time, expressed in seconds, is determined as the total duration of pause segments (to be noticed is that we have eliminate the initial and final pauses prior to assessment).

##### Prosody Analysis

The speech prosody assessment was performed in this work in terms of the mean and standard deviation of the signal intensity (*I*) and fundamental frequency (*f*_0_). 

##### Time-Domain Analysis

We performed a time-domain speech analysis targeting the assessment of signal intensity and periodicity, i.e., zero-crossing-based features [55]. 

The time-domain features targeted in this work and considered relevant for the assessment of speech intensity are the mean absolute value (*mav*), energy (*enrg*), and root mean square (*rms*), which are defined as follows:

(30) $$mav_{k}=\frac{1}{n}\sum_{i=1}^{n}\left|x_{i}\right|,$$

(31) $$enrg_{k}=\frac{1}{n}\overset{n}{\underset{i=1}{\sum }}sig_{i}^{2},\;k=\bar{1,nw},$$

(32) $$rms_{k}=\sqrt{\frac{1}{n}\sum_{i=1}^{n}sig_{i}^{2}},\;k=\bar{1,nw},$$

where *k* is the segment index, *n* is the segment length (in samples), and *nw* is the total number of segments [56].

The time-domain features targeted in our work and considered relevant for speech periodicity are the zero-crossing rate (*ZC*) and slope sign changes (*SSCs*), which are defined as follows:

(33) $$ZC_{k}=\sum_{i=2}^{n}\left(sgn\left(sig_{i-1}\cdot sig_{i}\right)=-1\right),k=\bar{1,nw},$$

(34) $$SSC_{k}=\overset{n}{\underset{i=3}{\sum }}\left(sgn\left(\left(sig_{i-1}-sig_{i-2}\right)\cdot \left(sig_{i}-sig_{i-1}\right)\right)=-1\right),k=\bar{1,nw},$$

where *k* is the segment index, *n* is the segment length (in samples), and *nw* is the total number of segments [56].

##### Frequency-Domain Analysis

We performed a frequency-domain speech analysis targeting the assessment of the power spectrum components and power spectrum shape [37]. The power spectrum (P) was generated for each 20 ms signal frame, and the frequency-domain features were extracted as follows.

The frequency-domain features targeted in this work for the assessment of the power spectrum components are the frequency of the maximum spectral component (*maxf*) and the weighted average of the frequency components (*waf*), defined as follows:

(35) $$maxf_{k}=\left\{f|P_{k}\left(f\right)=max\left(P_{k}\right)\right\},k=\bar{1,nw},$$

(36) $$waf_{k}=\frac{\sum_{i=1}^{n}P_{k}^{2}\left(f_{i}\right)\cdot f_{i}}{\sum_{i=1}^{n}P_{k}^{2}\left(f_{i}\right)},k=\bar{1,nw},$$

where *k* is the segment index, *n* is the segment length (in samples), and *nw* is the total number of segments. Note that, while the pitch is also a relevant power spectrum component assessment feature [25,26,37], it was previously addressed in a prosody assessment. 

The frequency-domain features targeted in this work for the assessment of the power spectrum shape are skewness and kurtosis [57]. 

##### LPC Analysis

The formants are estimated by means of the linear predictive coding (LPC) analysis. The first three formants (*f*_1_, *f*_2_, and *f*_3_) were considered for assessment in this work. 

The LPC analysis was preceded by a down-sampling of the speech signal from 44.1 kHz to 16 kHz and segmentation with a 2 ms rectangular widow with 50% overlap. A finer resolution was required, in comparison to the time-domain and frequency-domain analyses, to catch the vowels within the utterings and perform the formant analysis accordingly. 

#### 2.2.3. CNN-Based Spectrogram Classification

In this paper, convolutional neural networks (CNNs) were used to train data in order to classify speech into PD and HC classes. The CNN is a subdomain of AI that has achieved immense success in recent years. These neural networks are deep because their architecture is more complex and consists of several layers of convolution, providing an improvement in model performance with the increase of the dataset [46]. Using CNN, the extraction of features from images is performed automatically, and there is no need for human intervention. Therefore, convolutional networks have the role of recognizing certain characteristics from the images applied to the input of the model, based on the convolution operations, and recombining the features extracted in the final layers of the architecture to achieve the classification. Thus, the CNN improves the structure and performance of traditional artificial networks, and the architecture of these models is suitable for recognizing certain patterns, i.e., features from the structure of 2D images [47]. As a mode of use, the CNN achieved very good results in the analysis of medical images, image segmentation, or in the field of visual recognition [48]. 

CNN-based classification for the discrimination of Parkinsonian speech is performed in our work on spectrograms. The spectrogram is a three-dimensional plot of the signal amplitude vs. time and frequency [58] and can be employed for CNN-based classification [59]. Our motivation for spectrogram employment resides in the fact that it contains a visual representation of the Parkinsonian speech characterization features defined in Section 2.2.2. As such, we expect that the CNN-based classification of the speech spectrograms captures the feature-based Parkinsonian speech assessment. 

**Figure 4:**
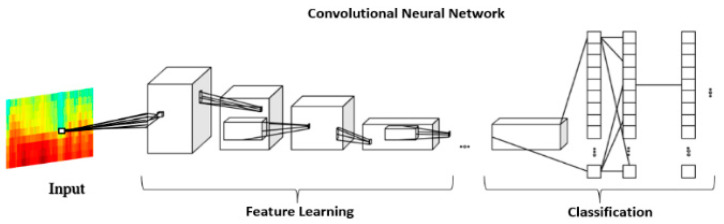
Workflow of the CNN-based classification of spectrograms aiming for the identification of Parkinsonian speech.

The CNN-based spectrogram classification workflow is illustrated in Figure 4. First, spectrograms of the speech sequences extracted from the VAD were generated. The spectrograms were saved as jpeg images and were applied to the CNN for speech classification. 

The MobilNet model is built on separable convolution, and all layers are followed by Relu activation functions, with the exception of the final layer, which is a complete convolution, and which is fully connected. The hyperparameter settings are listed in Table 4. The CNN structure is then given in Table 5.

Three types of spectrograms were used for CNN training: speech spectrograms, speech energy spectrograms, and vowel maps and Mel spectrograms.

The speech spectrogram provides a visual representation of the speech power spectrum variation in time. As such, the speech spectrogram can be used to assess the time-frequency amplitude distribution [58]. 

The speech energy spectrogram further provides a visual representation spectral energy distribution into short-term spectra on segments of speech. As such, the speech energy spectrogram tracks acoustic–phonetic changes [60]. 

Alternatively, the Mel spectrogram was derived as the short-term power spectrum, based on the linear cosine transformation of the log power spectrum, on a non-linear scale; provided a visual representation of the human hearing perception; and explored phonetic variation and change [61]. 

In our study, we used the MobileNet CNN architecture model. The MobileNet model performed feature extraction based on 28 layers of convolution, which are grouped into modules, offering a fast computation time [62], with the aim of maximizing accuracy and reducing the cost of computation [63]. MobileNet uses depth-wise separable convolutions to reduce the number of parameters and size of the model and tracks the balance between compression and precision.

The CNN model was trained in Google Colab, using Python. Our choice for the Colab programming environment was motivated by the free Graphics Processing Unit (GPU) services that allow the construction and automatic training of neural networks by performing parallel tasks on large datasets. Network training was performed with a learning rate of 0.005. That means the amount that the weights are updated during training is 0.5%. This is the most important parameter in the network training process, as it regulates its performance, controlling the rate at which the algorithm learns parameter values. Moreover, we chose to use the *batch_size* parameter set to 128 to use less memory during training and to speed up the training procedure. The number of epochs used for the complete training cycles of the networks is variable and is chosen between 100 and 200 epochs.

## 3. Results

### 3.1. Wiener Filter Performance Evaluation

The statistics of the estimated speech enhancement and fidelity measures are listed in Table 6. The complete record of the speech enhancement and fidelity measures, which were computed for every subject in the study group, is listed in Appendix A Table A1.

### 3.2. Feature Extraction for Parkinsonian Speech Assessment

The results of the feature extraction stages applied for phonology, prosody, time-domain, frequency-domain, and formant analyses are described as follows.

#### 3.2.1. Phonological Analysis

The phonological speech parameters assessed in this work are expressed in terms of uttering count, pause count, speech rate, and pause duration.

**Figure 5:**
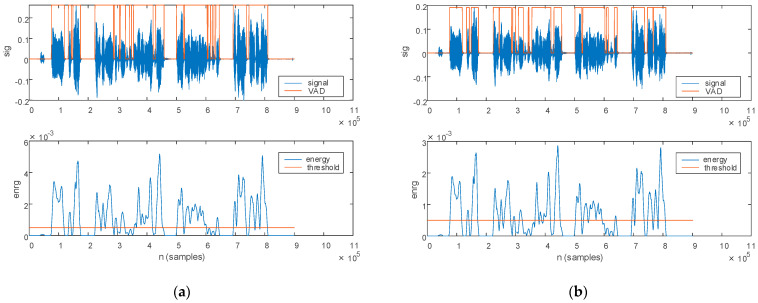
The voice activity detection procedure illustrated for a PD patient: (**a**) original signal and (**b**) filtered signal. The top figure plots the signal (blue) and the detected voice activity (orange). The bottom figure plots the signal energy (blue) and the comparison threshold (orange).

The first stage in phonology assessment assumes the discrimination of utterings from pauses. The energy-based VAD described in Section 2.2 is employed for this purpose. The results of the voice activity detection procedure are depicted in Figure 5 for a PD patient. The original speech sample with the corresponding signal energy is plotted in Figure 5a, and the filtered speech sample with the corresponding signal energy is plotted in Figure 5b. 

The comparison threshold, plotted with orange on the energy plot, is set empirically to 1/10 of the maximum signal energy. Utterings are then identified for signal energy levels above the comparison threshold, as plotted with orange on the speech sample.

As illustrated in Figure 5, noise in the original signal leads to different energy values in contrast to the filtered signal. The identification of utterings and pauses thus leads to different results on the two signals. Consequently, the phonological parameters estimated from the VAD are also different for the original and filtered signal.

**Figure 6:**
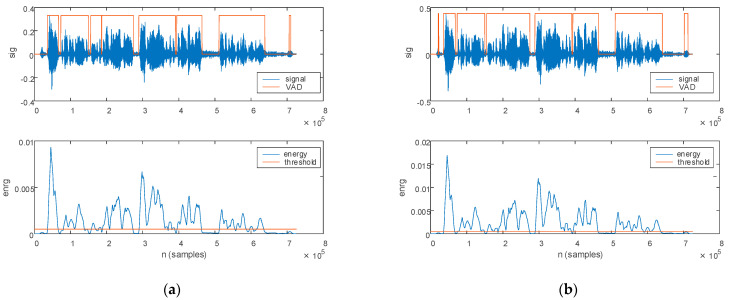
The voice activity detection procedure illustrated for an HC: (**a**) original signal and (**b**) filtered signal. The top figure plots the signal (blue) and the detected voice activity (orange). The bottom figure plots the signal energy (blue) and the comparison threshold (orange).

The same voice activity detection procedure is depicted for an HC in Figure 6. The original signal with the corresponding signal energy are plotted in Figure 6a. The filtered signal with the corresponding signal energy is plotted in Figure 6b. 

The uttering count and the pause count were determined directly from the voice activity detection results. The VAD further enables the assessment of the speech rate and pause duration on the entire speech sample. Statistics of the extracted phonological parameters, namely *n_uttering_*, *n_pause_*, *r_speech_*, and *t_pause_*, are listed in Table 7 for both original and filtered speech samples. The complete record of the phonological features, which were computed for every subject in the study group, is listed given in Appendix A Table A2.

#### 3.2.2. Prosody Analysis

**Figure 7:**
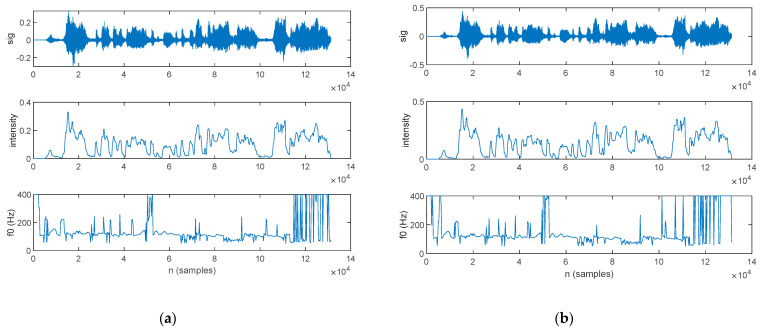
The prosody features extracted for a PD patient: (**a**) original signal and (**b**) filtered signal. The top figure plots the speech sample, the middle figure plots the signal intensity, and the bottom figure plots the pitch.

The prosody features are evaluated in this work in terms of speech intensity (*I*) and pitch, i.e., fundamental frequency (*f*_0_). The prosody features computed on the speech sample of a PD patient are plotted in Figure 7, with Figure 7a illustrating the features estimated from the original signal, and Figure 7b from the filtered signal.

**Figure 8:**
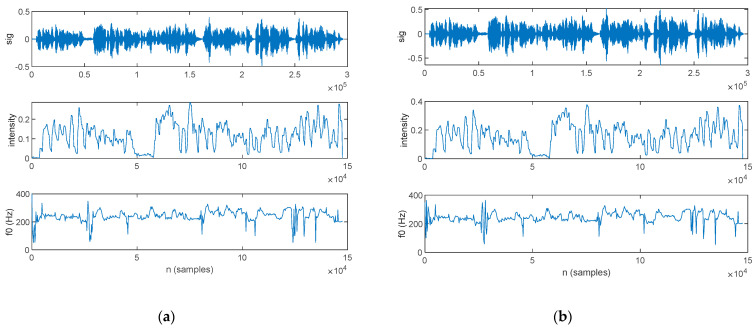
The prosody features extracted for an HC: (**a**) original signal and (**b**) filtered signal. The top figure plots the speech sample, the middle figure plots the signal intensity, and the bottom figure plots the pitch.

The prosody features computed on the speech sample of an HC are plotted in Figure 8, with Figure 8a illustrating the features estimated form the original signal and Figure 8b from the filtered signal.

We estimated the mean (*µ*) and standard deviation (*σ*) of the prosody speech parameters. The statistics of the extracted speech prosody, in mean and standard deviation, are listed in Table 8 for both the original and filtered speech samples. Note that the fundamental frequency metrics are assessed separately for the male and female subjects. The complete record of the prosody features, computed for every subject in the study group, is listed in Appendix A Table A3.

#### 3.2.3. Time-Domain Analysis

The time-domain features determined in this work are the intensity-based features, i.e., MAV, E and RMS; and the periodicity-based features, i.e., *ZC* and *SSC*. 

**Figure 9:**
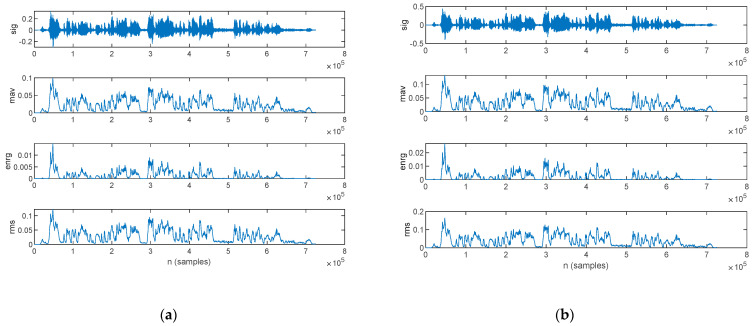
The time-domain intensity-based features extracted for a PD patient: (**a**) original signal and (**b**) filtered signal. The top figure plots the speech sample, followed by the mean absolute value, signal energy, and root mean square.

The time-domain intensity-based features estimated from the speech sample of a PD patient are plotted in Figure 9: those for the original signal are shown in Figure 9a, and those from the filtered signal are in Figure 9b.

**Figure 10:**
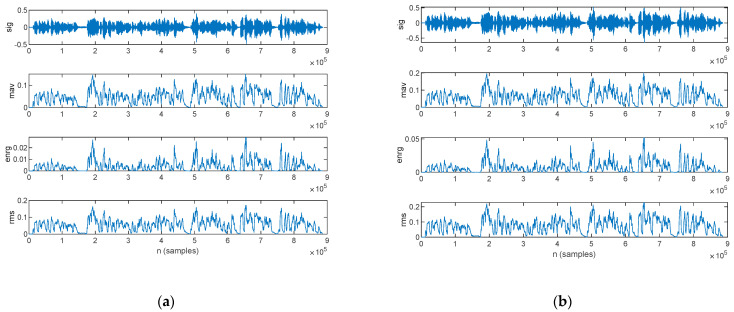
The time-domain intensity-based features extracted for an HC: (**a**) original signal and (**b**) filtered signal. The top figure plots the speech sample, followed by the mean absolute value, signal energy, and root mean square.

The time-domain intensity-based features estimated form the speech sample of an HC are plotted in Figure 10: those for the original signal are shown in Figure 10a, and those for the filtered signal are in Figure 10b.

The statistics for the time-domain intensity-based features, in mean value and standard deviation, are listed in Table 9 for both the original and filtered speech samples. The complete record of the intensity-based time-domain features, computed for every subject in the study group, is listed in Appendix A Table A4. 

**Figure 11:**
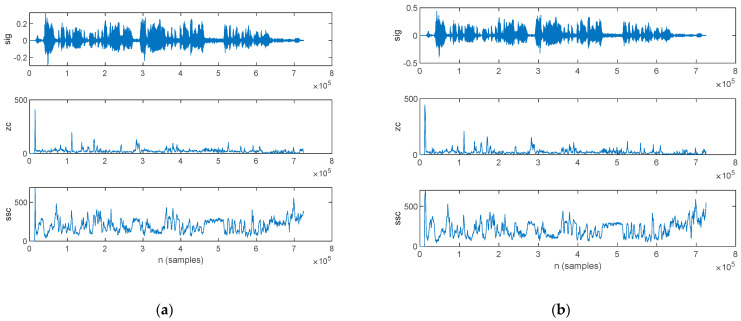
The time-domain periodicity-based features extracted for a PD patient: (**a**) original signal and (**b**) filtered signal. The top figure plots the speech sample, followed by the zero-crossing rate and slope sign changes.

The time-domain periodicity-based features estimated from the speech sample of a PD patient are plotted in Figure 11: those for the original signal in are shown in Figure 11a, and those for the filtered signal are in Figure 11b.

**Figure 12:**
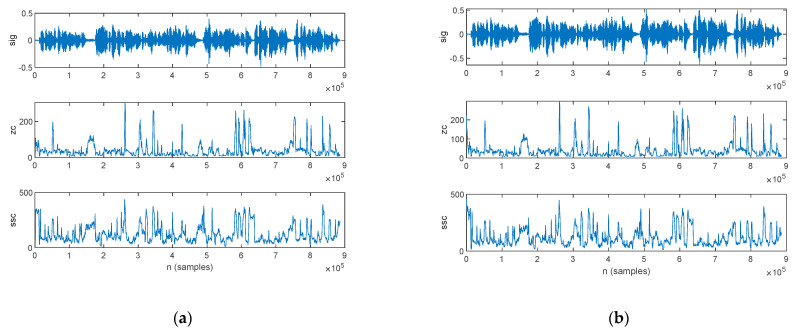
The time-domain periodicity-based features extracted for an HC: (**a**) original signal and (**b**) filtered signal. The top figure plots the speech sample, followed by the zero-crossing rate and slope sign changes.

The time-domain periodicity-based features estimated from the speech sample of an HC are plotted in Figure 12: those for the original signal are shown in Figure 12a, and those for the filtered signal are in Figure 12b.

The statistics for the time-domain periodicity-based features, in mean value and standard deviation, are listed in Table 10 for the both original and filtered speech samples. The complete record of the periodicity-based time-domain features, computed for every subject in the study group, is listed in Appendix A Table A5.

#### 3.2.4. Frequency-Domain Analysis

The frequency-domain features determined in this work for the power spectrum assessment are MAXf and WAF. The frequency-domain features which assess the power spectrum shape are expressed in terms of skewness and kurtosis. 

**Figure 13:**
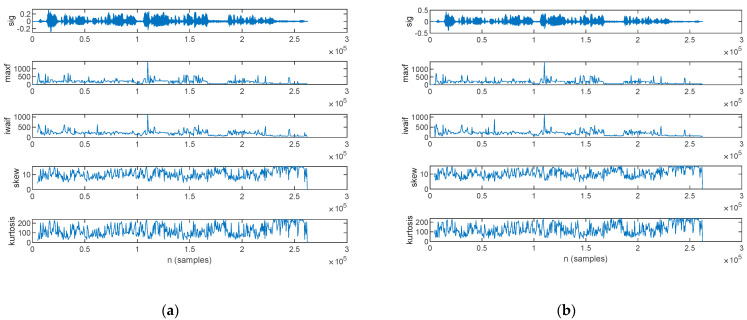
The frequency-domain features extracted for a PD patient: (**a**) original signal and (**b**) filtered signal. The top figure plots the speech sample, followed by the frequency of the maximum spectral component, weighted average of the frequency components, skewness, and kurtosis.

The frequency-domain features estimated from the speech sample of a PD patient are plotted in Figure 13: those for the original signal are shown in Figure 13a, and those for the filtered signal are in Figure 13b.

**Figure 14:**
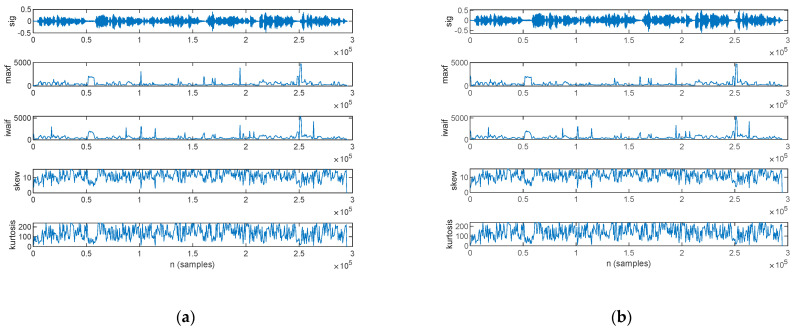
The frequency-domain features extracted for an HC: (**a**) original signal and (**b**) filtered signal. The top figure plots the speech sample, followed by the frequency of the maximum spectral component, weighted average of the frequency components, skewness, and kurtosis.

The frequency-domain features estimated from the speech sample of an HC are plotted in Figure 14, for the original signal in Figure 14a and the filtered signal in Figure 14b.

The statistics of the frequency-domain features, in mean value and standard deviation, are listed in Table 11 for both the original and filtered speech samples. The complete record of the frequency-domain features, computed for every subject in the study group, is listed in Appendix A Table A6 for the mean value and Table A7 for the standard deviation.

#### 3.2.5. LPC Analysis

**Figure 15:**
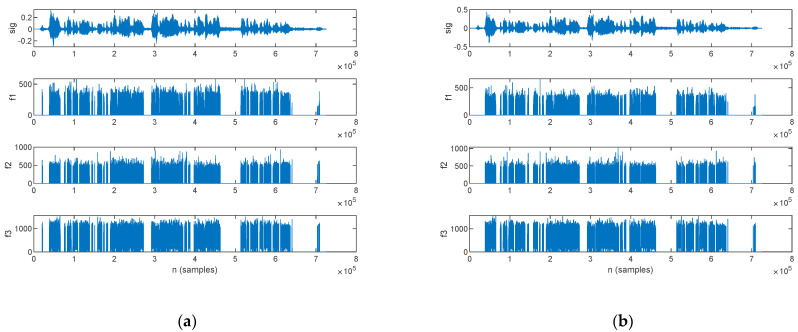
The speech sample (top) and the first three formants (*f*_1_, *f*_2_, and *f*_3_) extracted for a PD patient: (**a**) original signal and (**b**) filtered signal.

An LPC analysis was performed in this work, with the aim of formant extraction. The first three formants extracted for a PD patient are plotted alongside the speech sample in Figure 15: those for the original signal are shown in Figure 15a, and those for the filtered signal are in Figure 15b.

**Figure 16:**
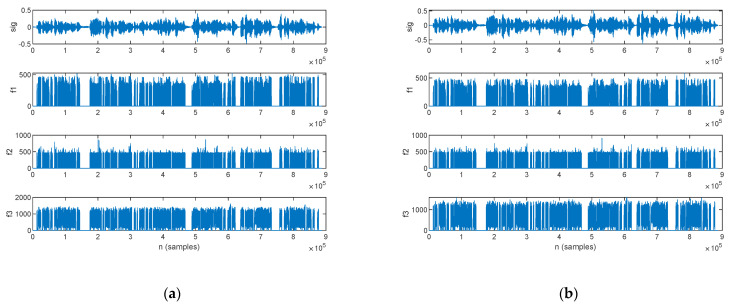
The speech sample (top) and the first three formants (*f*_1_, *f*_2_, and *f*_3_) extracted for an HC: (**a**) original signal and (**b**) filtered signal.

The first three formants extracted for an HC are plotted alongside the speech sample in Figure 16; those for the original signal are shown in Figure 16a, and those for the filtered signal are in Figure 16b.

The statistics of the first three formants, in mean value and standard deviation, are listed in Table 12 for both the original and filtered speech samples. The complete record of the formants, which were computed for every subject in the study group, is listed in Appendix A Table A8 for the mean value and Table A9 for the standard deviation.

### 3.3. CNN-Based Spectrogram Classification

**Figure 17:**
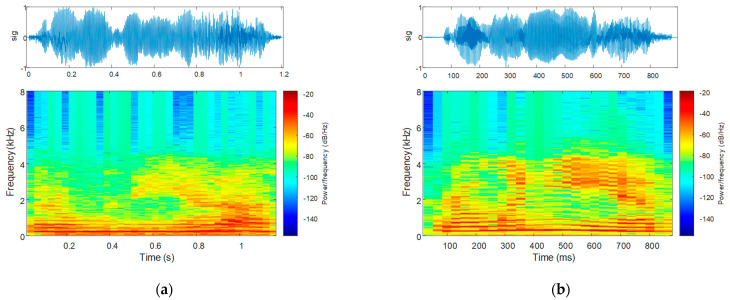
The speech sample (top) and the speech spectrogram (bottom) for the uttering of the word “Românie” by (**a**) a PD patient and (**b**) an HC.

The speech spectrogram of the sequence corresponding to the uttering of the word “Românie” in Romanian language, consisting of four vowels—two individual vowels and one vowel group—is plotted alongside the waveform of the uttering in Figure 17: that for a PD patient is shown in Figure 17a, and that for an HC is in Figure 17b.

**Figure 18:**
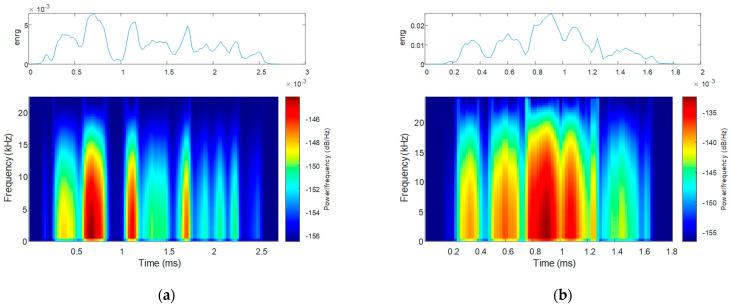
The speech energy (top) and the speech energy spectrogram (bottom) for the uttering of the word “Românie” by (**a**) a PD patient and (**b**) an HC.

The speech energy spectrogram corresponding to the uttering of the same word is plotted in Figure 18, alongside the waveform of the uttering: that for a PD patient is shown in Figure 18a, and that for an HC is in Figure 18b.

**Figure 19:**
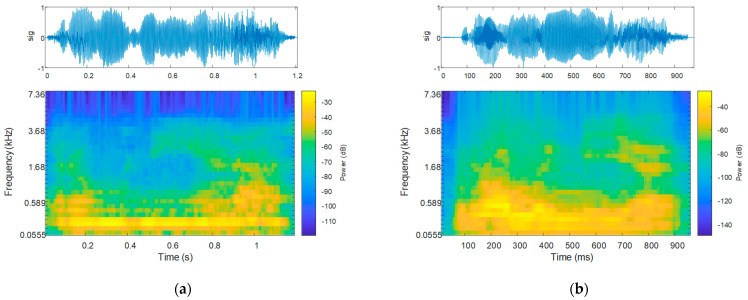
The speech sample (top) and the Mel spectrogram (bottom) for the uttering of the word “Românie” by (**a**) a PD patient and (**b**) an HC.

The Mel spectrogram of the sequence corresponding to the uttering of the same word is plotted in Figure 19, alongside the waveform of the uttering: that for a PD patient is shown in Figure 19a, and that for an HC is in Figure 19b.

The dataset for the CNN consists of the spectrograms for the speech sequences extracted from the speech samples of the 27 subjects: 16 patients diagnosed with PD and 11 healthy controls. Accordingly, the dataset for the original speech samples consists of 318 utterings: 215 for PD patients and 103 for HCs. The dataset for the filtered speech samples consists of 289 utterings: 194 for PD patients and 95 for HCs. The dataset was divided into the training dataset—accounting for 80%, with 20% used for validation; and the test dataset—accounting for 20%.

The classification accuracy was evaluated according to accuracy (acc) and loss [64,65]. Accuracy is defined as

(37) $$\mathrm{acc}=\frac{\mathrm{TP}+\mathrm{TN}}{\mathrm{TP}+\mathrm{TN}+\mathrm{FP}+\mathrm{FN}},$$

with the parameters accounting for true positives (TPs), true negatives (TNs), false positives (FPs), and false negatives (FNs). The TP and TN metrics count the correct classifications, whereas the FP and FN metrics count the incorrect classifications. Accordingly, the accuracy indicates the probability of accurately identifying the samples in either of the two classes. Loss, on the other hand, is an indicator of the deviation between the predicted values and the real labels. Binary cross entropy is a commonly used loss function in binary classification problems. It measures the difference between the predicted probabilities and the true labels for each data point. Moreover, binary cross entropy has a probabilistic interpretation: it can be viewed as the negative log likelihood of the true label under the predicted probability distribution. In other words, the lower the loss, the higher the likelihood that the model’s predictions are correct. Overall, binary cross entropy is a good choice for binary classification tasks because it is easy to compute, has a probabilistic interpretation, and can be optimized efficiently by using gradient-based methods.

The estimated CNN performance metrics that were obtained after network training, in terms of accuracy FP, FN, and loss, are listed in Table 13. As illustrated, the best results were obtained based on speech energy, with an accuracy of 96% and a loss of only 0.12. Speech spectrograms and Mel spectrograms led to lower accuracy values.

A closer inspection of the speech phonological parameters, which are given in Appendix A Table A2, points out that the patients PD 1, PD 4, PD 5, PD 11, and PD 13 exhibit feature values in the HC range, contradicting the guidelines prescribed by Boschi et al. [25]. Contrarywise, the healthy controls HC 4, HC 5, and HC 8 exhibit feature values in the PD range. 

Thus, in the second CNN training attempt, we eliminated the speech spectrograms of the subjects with feature values outside the variation range prescribed by the statistics reported in Table 6 and Table 7. In this case, the CNN dataset for the original speech samples is reduced to 241 utterings: 181 for PD patients and 60 for HCs. The dataset for the filtered speech samples is reduced to 222 utterings: 166 for PD patients and 56 for HCs. The classification accuracy, however, is improved, becoming 93%, in the case of the filtered signal, with a loss of only 0.1. The dataset distribution for CNN training and validation is the same.

The classification accuracy achieved in this work is listed in comparison to values reported in the literature in Table 14 and Table 15. Table 14 points out that the classification accuracy depends primarily on the speech task. Sustained vowel phonation and diadochokinetic tasks account for phonetic segment duration in the order of magnitude of seconds. In extremis, [13] reported on sustained vowel phonation with a duration of 2 s. Thus, feature extraction provides a good feature resolution, and consequently, there are sufficient numeric data available for assessment and classification. This makes vowels and diadochokinetic tasks appropriate for classification using supervised learning architectures such as k-NN, SVM, or RF. Contrarywise, phonetic segments in the continuous speech samples are limited to 100–300 ms [66]. In such cases, the feature resolution is rather small; thus, neural network architectures are more suitable for classification.

Sustained vowel phonation and diadochokinetic tasks reach large classification accuracy values. Specifically, the highest classification accuracies were achieved for sustained vowel phonation in [13,39]. Table 14 points out that we were able to report comparable accuracy values. On the other hand, there is only a small number of solutions in the literature which report on Parkinsonian speech identification from continuous speech, and which also reach lower classification accuracies [38,43,44]. From this point of view, the classification accuracy reported in our work is larger than the accuracy reported in the literature for a similar task. 

Furthermore, the speech samples classified in our study were recorded in-clinic, an inherently noisy environment, in contrast to a soundproofed laboratory environment, as was the case in the related work. 

With respect to the aim of our study, which targeted the CNN-based identification of PD from continuous speech, we compared our results to others obtained using deep learning models. As illustrated in Table 15, the classification accuracy we achieved in our study using CNNs is higher than the accuracy reported in [38,44]. On the other hand, the larger accuracy reported in [13] was achieved on sustained vowel phonation, in contrast to running speech, which was the case in our work.

## 4. Discussion

### 4.1. Speech Enhancement and Fidelity Measures

The SNR values indicate a clear improvement of the speech samples with Wiener filtering. As a quantitative measure of the signal improvement, the SNRI indicates that Wiener filtering improved the speech signal with an average 4 dB for both PD patients and HC. The MSE in the10^−4^ order of magnitude indicates that there are no severe deviations between the original and fileted speech signals. It is thus sensible to assume that relevant information for the characterization of Parkinsonian speech was not lost with filtering.

### 4.2. Feature Extraction for Parkinsonian Speech Assessment

#### 4.2.1. Phonology Analysis

The phonological features extracted from the speech samples confirm previous results reported by Boschi et al. as relevant [25]. Accordingly, our results illustrate that Parkinsonian speech exhibits an increased pause count in comparison to HCs, which is consistent with hypokinetic phonation and voice blocking [18]. The total pause duration, attributable to inappropriate silence [18], is also larger for PD patients. 

Furthermore, uttering count and speech rate—estimated in our study as the number of utterings per minute, exhibit larger values for PD patients. This result is attributable to the dysfluent nature of speech in PD [18,33]. 

With respect to filtering, although the specific feature values were changed, the feature relationships hold for both original and filtered speech samples. 

#### 4.2.2. Prosody Analysis

Our results on prosody assessment exhibit smaller values for speech intensity, in both mean and standard deviation, for PD in comparison to HC. While the smaller mean reveals reduced voice intensity and speech loudness, the smaller standard deviation reveals the mono-loudness attribute of Parkinsonian speech. 

The standard deviation of the fundamental frequency, reported in the literature as an indicator for intonation-related impairment [27,31], reveals a smaller value in the case of Parkinsonian speech. 

The effects of Wiener filtering on the prosody features of speech accounts for changes in the intensity mean and standard deviation values, because of noise suppression. The differences in the fundamental frequency are insignificant. Nevertheless, the relationship between the prosody features holds for both original and filtered speech samples.

#### 4.2.3. Time-Domain Analysis

The time-domain analysis of the speech samples illustrates that the intensity-based features are smaller for Parkinsonian speech in comparison to HC, in both mean and standard deviation. This relationship is consistent with the attributes of Parkinsonian speech [28]. Indeed, smaller mean values are an indicator of reduced voice intensity and speech loudness. Smaller standard deviation values are an indicator for mono-loudness speech and reduced intensity modulation. These relationships hold for both original and filtered speech samples; the difference in feature values is, however, more pronounced for the filtered signal.

The periodicity-based features exhibit a smaller zero-crossing rate value for Parkinsonian speech in comparison to HC, in both mean value and standard deviation. This result is consistent with the mono-pitch attribute of Parkinsonian speech [28]. Slope sign changes, on the other hand, exhibit a larger value for Parkinsonian speech, in both mean value and standard deviation. These relationships hold for both original and filtered speech samples; yet again, the difference in feature values is more pronounced for the filtered signal.

#### 4.2.4. Frequency-Domain Analysis

A frequency-domain analysis was performed in this work to assess the spectral content by means of the maximum component frequency and the weighted average of the frequency components. Further on, the spectrum shape was assessed by means of skewness and kurtosis.

Our assessment results show that both power spectrum component features are lower for Parkinsonian speech in comparison to HC. The lower maximum component frequency of Parkinsonian speech originates from breathy voice [60] and indicates that breath is the dominant speech component in the presence of reduced voice intensity. The lower weighted average of the frequency components, on the other hand, provides a numeric estimate which captures phonation, expressivity, modulation, and articulation difficulties [28,31]. These relationships stand for both original and filtered speech sequences. 

Spectrum shape assessment exhibits a similar skewness value for PD and HC, whereas kurtosis exhibits larger values for Parkinsonian speech. The difference in kurtosis, however, is small, and we cannot base the discrimination of Parkinsonian speech on this feature. Wiener filtering does not change the spectrum shape feature values.

#### 4.2.5. LPC Analysis

A formant analysis addresses the assessment of incorrect articulation as a characteristic of Parkinsonian speech [18,31,33]. Indeed, *f*_1_ is produced by jaw movement, whereas *f*_2_ is produced by tongue movement [67]. In this work, we performed formant extraction by means of an LPC analysis.

Our assessment results show that the standard deviation of the formants is smaller for parkinsonian speech in comparison to HC. Considering that we performed the assessment on samples of continuous speech, this result is accountable to imprecise articulation of consonants [18] and is consistent with hypokinetic speech. 

These relationships hold for both original and filtered speech samples; moreover, filtering does not change the formant frequencies significantly.

### 4.3. CNN-Based Spectrogram Classification

Three types of spectrograms were employed in this work for CNN-based speech classification: speech spectrograms, speech energy spectrograms, and Mel spectrograms. We argue that several features of Parkinsonian speech, identified with prosody, time-domain, frequency-domain, and LPC analyses, are contained in these spectrograms. This was our motivation for spectrogram employment in the CNN-based classification of Parkinsonian speech. 

The speech spectrogram, as a representation of the speech intensity in the time-frequency coordinate system [58], visualizes reduced voice intensity and speech loudness in PD. Furthermore, the speech spectrogram visualizes relatively constant spectral maxima vs. time in PD. As discussed for the feature assessment, these attributes are consistent with Parkinsonian softness of voice, reduced speech modulation, articulation, and expressivity [18,27,28,31,31]. Furthermore, the speech spectrograms provide a better visualization of breathy voice [60].

Reduced speech loudness of the PD patient in contrast to the HC is also visible in the speech energy and Mel spectrograms. The speech energy spectrogram further visualizes acoustic–phonetic changes [60], which are more abrupt in the case of the PD patient. 

Both speech and Mel spectrograms visualize that the energy content in the case of Parkinsonian speech is confined to smaller frequencies in contrast to HCs. However, this is more pronounced on the Mel spectrogram, which highlights a spectral peak that stays constant vs. time. This is consistent with the mono-pitch attribute of Parkinsonian speech [28].

Feature-based speech assessment points out that certain patients exhibit phonological feature values in the HC range, whereas certain healthy controls exhibit feature values in the PD range. This observation is extrapolated to the spectrogram analysis. As such, we attempted to eliminate from the dataset all speech spectrograms generated for subjects with phonological feature values outside the specified variation ranges. The classification accuracy on speech spectrograms was improved from 78% with 0.3 loss to 85% with 0.8 loss for the unfiltered signals and from 86% with 0.4 loss to 95% with 0.1 loss on the filtered signals. The classification accuracy on speech energy spectrograms was improved from 80% with 0.3 loss to 87% with 0.4 loss for the unfiltered signals and from 84% with 0.6 loss to 96% with 0.1 loss on the filtered signals. The classification accuracy on Mel spectrograms was improved from 58% with 0.5 loss to 87% with 0.7 loss for the unfiltered signals and from 70% with 0.3 loss to 92% with 0.5 loss on the filtered signals. As illustrated, our approach led to the improvement of classification accuracy.

The highest accuracy improvement achieved on Mel spectrograms is motivated by the fact that Mel spectrograms visualize speech perception [60]. Thus, it is inferable that the speech samples which are assessed with a feature-based analysis to be healthy also account for the perception of the speech sample to be healthy. 

Regarding noise suppression, the 4 dB SNR improvement achieved with the Wiener optimal filter on the speech samples produces an improvement in the CNN-based classification accuracy of 8–12%. Indeed, as a result of noise suppression, the spectrograms only contain relevant speech information. 

The best CNN-based PD classification accuracy was achieved for the speech energy spectrograms, both before and after data set reduction and regardless of filtering. This result is explained by the fact that the speech energy spectrogram captures acoustic–phonetic changes on segments of speech [60] for which PD is identifiable [31]. 

Regarding our choice for the MobileNet model, it is mainly based on our previous study in [64], wherein we investigated the MobileNet, EfficientNet and Xception models for image classification in the discrimination of PD. Since we obtained the best classification accuracy with the MobileNet, it was our straightforward choice for the present study. 

### 4.4. Limitations

In this paper, we analyzed phonological features, prosody features, time-domain features, frequency-domain features, and LPC analysis for formant extraction. The reported features measure the Parkinsonian traits of continuous speech, confirming the particularities of PD vs. HC in terms of loudness, intonation, phonation, prosody, and articulation.

Given the continuous nature of the speech task, the duration of the voiced segments is considerably shorter than for sustained vowel phonation and diadochokinetic tasks. Specifically, we can only isolate vowels with a duration of 100–200 ms vs. sustained vowel phonation, which accounts for 2 s in duration [13]. As such, a limitation of our work is that we are unable to assess feature standard deviations attributable to tremor phonation on voiced segments. Specifically, while the standard deviation of pitch, energy, and formants on vowel phonation and diadochokinetic tasks is reported to be larger for PD in comparison to HC [13,25], we report on larger values for HC, and this is attributable to voice modulation, expressivity, and articulation throughout the continuous speech. 

With regard to speech sample recording in noisy environment, we confirmed that the Wiener optimal filter is applicable for noise suppression, while maintaining the Parkinsonian speech attributes. However, the limitations of Wiener filtering in the presented application occur when the speech is recorded with background talk, hospital traffic, etc., which is interpreted by the filter as voice activity rather than noise, and therefore, it is not suppressed. 

## 5. Conclusions

In this paper, we discussed AI-based identification of Parkinsonian speech. The novelty of this work is twofold. First, we performed Parkinsonian speech assessment on samples of continuous speech. Second, we recorded the speech samples in the clinic, in an inherently noisy environment, and thus we were able to analyze and quantify the Wiener filter’s applicability to speech denoising for the identification of Parkinsonian speech. We concluded that Wiener filter improves both feature-based-analysis and CNN-based-classification performances. 

The proposed speech assessment methodology for the AI-based identification of Parkinsonian speech follows speech acquisition, processing, feature extraction, feature assessment, and finally CNN-based classification of spectrograms generated from the speech samples. Our target was to assess loudness, intonation, phonation, prosody, and articulation of speech by means of phonological, prosody, time-domain, frequency-domain, and LPC features respectively. We argue that the Parkinsonian traits identified with the feature-based speech analysis are contained in the spectrograms. Then, the best classification accuracies we achieved were 96% on speech energy, 93% on speech, and 92% on Mel spectrograms.

The assessment results reported in this paper confirm the results previously reported in the literature. Nevertheless, the strength of our results is that we achieved them on samples of continuous speech rather than short speech segments, e.g., sustained vowels, short syllables/words, or short sentences. Furthermore, the speech samples used for the Parkinsonian speech assessment and CNN training were acquired from patients and healthy controls in our targeted study group, following a research protocol that we devised ourselves, and not from publicly available third-party speech databases where we have no control over the acquisition and processing protocol. 

The results reported in this paper can constitute guidelines for a running speech assessment methodology in PD. This could lay down the foundation for new applications to assess the quality of spoken communication. 

Our future research is oriented towards the development of an autonomous AI-based decision support system for PD pre-diagnosis. We aim to integrate the methodology proposed and developed in this study, along with our previously reported solutions on the tremor [45], gait [64,68], and written communication assessment [45], in correlation with Parkinson’s disease rating scales, cognitive evaluation, and the resulting socioeconomic impact.

## Figures and Tables

**Table 1:** Feature classes, categorized by the speaking task, for the objective assessment and identification of hypokinetic dysarthria manifestations.

Hypokinetic Dysarthria Manifestation	Speaking Task				
	Sustained Vowel Phonation	Diadochokinetic Task	Isolated Words	Short Sentences	Continuous Speech
Voice blocking	n.a.	n.a.	n.a.	Phonology	Phonology
Mono-pitch oration	n.a.	n.a.	n.a.	n.a.	MFCCs
Mono-loudness oration	n.a.	n.a.	n.a.	n.a.	MFCCs
Tremor phonation	Prosody	Prosody	Prosody	Prosody	MFCCs
Voice quality	Time domain Frequency domain	Time domain Frequency domain	Time domain Frequency domain	Time domain Frequency domain	MFCCs
Impaired articulation	Formants	Formants	Formants	n.a.	MFCCs

n.a.—not available/not reported. MFCCs—Mel-frequency cepstral coefficients.

**Table 2:** Parkinsonian speech assessment features, categorized by the feature classes.

Feature Class	SNRI	Reference
Phonology	Speech and silence statistics: speech rate, number of pauses, pause duration, phonemic errors, phonation time, locution time, filled pauses, false starts	[25,26]
Prosody	Pitch	[27,28]
	*σ*(*f*_0_), *σ*(*I*)	[13,25,26,27,29,30,31]
	HNR	[26,32]
	Shimmer, jitter	[26]
Time domain	Energy	[37]
	Zero-crossing rate	[37]
Frequency domain	Filter bank energy coefficient, spectral sub-band centroid	[26]
	Skewness, kurtosis	[37]
Formants	*f*_1_, *f*_2_, *f*_3_	[13,31,33,34,36]
MFCC	MFCC	[26,35,38]
	Derivatives of the MFCC	[38]

**Table 3:** Parkinsonian speech assessment features targeted in this work.

Feature Set	SNRI
Phonology	Uttering count (*n_uttering_*), number of pauses (*n_pause_*), speech rate (*r_speech_*), pause duration (*t_pause_*)
Prosody	Intensity (*I*), fundamental frequency (*f*_0_)
Time domain	Mean absolute value (*mav*), energy (*enrg*), root mean square (*rms*), zero-crossing rate (*ZC*), slope sign changes (*SSC*)
Frequency domain	Frequency of the maximum spectral component (*maxf*), weighted average of the spectral components (*waf*), skewness, kurtosis
Formants	*f*_1_, *f*_2_, *f*_3_

**Table 4:** The CNN hyperparameter settings.

Hyperparameter	Value
Learning rate	0.005
Loss function	BinaryCrossentropy
Activation function	RELU
Batch normalization	active
Epochs	100
Data augmentation	RandomContrast (factor = 0.3)
	RandomFlip (mode = “horizontal”)
	RandomRotation (factor = 0.18)

**Table 5:** The CNN structure.

Type/Stride		Filter Shape	Input Size
Conv/s2		3 × 3 × 3 × 32	224 × 224 × 3
Conv dw/s1		3 × 3 × 32 dw	112 × 112 × 32
Conv/s1		1 × 1 × 32 × 64	112 × 112 × 32
Conv dw/s2		3 × 3 × 64 dw	112 × 112 × 64
Conv/s1		1 × 1 × 64 × 128	56 × 56 × 64
Conv dw/s1		3 × 3 × 128 dw	56 × 56 × 128
Conv/s1		1 × 1 × 128 × 128	56 × 56 × 128
Conv dw/s2		3 × 3 × 128 dw	56 × 56 × 128
Conv/s1		1 × 1 × 128 × 256	28 × 28 × 128
Conv dw/s1		3 × 3 × 256 dw	28 × 28 × 256
Conv/s1		1 × 1 × 256 × 256	28 × 28 × 256
Conv dw/s2		3 × 3 × 256 dw	28 × 28 × 256
Conv/s1		1 × 1 × 256 × 512	14 × 14 × 256
5×	Conv dw/s1 Conv/s1	3 × 3 × 512 dw 1 × 1 × 512 × 512	14 × 14 × 512 14 × 14 × 512
Conv dw/s2		3 × 3 × 512 dw	14 × 14 × 512
Conv/s1		1 × 1 × 512 × 1024	7 × 7 × 512
Conv dw/s2		3 × 3 × 1024 dw	7 × 7 × 1024
Conv/s1		1 × 1 × 1024 × 1024	7 × 7 × 1024
Avg Pool/s1		Pool 7 × 7	7 × 7 × 1024
FC/s1		1024 × 1000	1 × 1 × 1024
Softmax/s1		Classifier	1 × 1 × 1000

**Table 6:** Statistics of the Wiener filter speech enhancement and fidelity measures.

Feature	Original Signal		Filtered Signal	
	PD	HC	PD	HC
SNR	39.3 ± 17.4	34.7 ± 8.6	43.5 ± 16.5	39.3 ± 8.9
SNRI	-	-	4.1 ± 2.6	4.6 ± 2.3
MSE	-	-	(2.8 ± 2.2) × 10^−4^	(5.1 ± 2.8) × 10^−4^

**Table 7:** Statistics of the phonological parameters.

Feature	Original Signal		Filtered Signal	
	PD	HC	PD	HC
*n_uttering_*	13.9 ± 7.4	9.4 ± 4.1	12.6 ± 6.9	8.6 ± 3.5
*n_pause_*	12.9 ± 7.4	8.4 ± 4.1	11.6 ± 6.9	7.6 ± 3.5
*r_speech_*	39.4 ± 8.3	31.6 ± 12.3	33.1 ± 9.8	28.6 ± 8.8
*t_pause_*	8.3 ± 7.9	4.6 ± 2.4	5.8 ± 3.2	4.3 ± 2.4

**Table 8:** Statistics of the speech prosody parameters, in mean and standard deviation.

Feature	Original Signal		Filtered Signal	
	PD	HC	PD	HC
*µ*(*I*)	72.8 ± 42.4	92 ± 16.5	78.2 ± 38.5	95 ± 21.3
*σ*(*I*)	88.3 ± 45.4	106 ± 13.3	95.3 ± 40.7	107.5 ± 12.7
*µ*(*f*_0_)	157.5 ± 39.8	174.5 ± 38.2	163.3 ± 40.4	176.2 ± 38.2
*σ*(*f*_0_)	59.5 ± 22.7	48.7 ± 23.2	60.4 ± 21.7	54.5 ± 18.5
*µ*(*f*_0_) male	138.8 ± 33.9	150.9 ± 17	145.3 ± 35.4	153.2 ± 18.1
*σ*(*f*_0_) male	49.8 ± 15.2	44.1 ± 22.1	54 ± 19	64.7 ± 18.9
*µ*(*f*_0_) female	188.6 ± 28.8	202.9 ± 38	193.2 ± 30.5	203.7 ± 38.8
*σ*(*f*_0_) female	75.8 ± 24.9	54.2 ± 25.8	71 ± 23.1	42.4 ± 8.8

**Table 9:** Statistics of the time-domain intensity-based features, in mean and standard deviation.

Feature	Original Signal		Filtered Signal	
	PD	HC	PD	HC
*µ*(*mav*)	36 ± 13	47 ± 13	47 ± 18	61 ± 18
*σ*(*mav*)	27 ± 13	34 ± 10	38 ± 18	46 ± 13
*µ*(*enrg*)	0.3 ± 0.3	0.5 ± 0.3	0.7 ± 0.6	1 ± 0.6
*σ*(*enrg*)	0.5 ± 0.4	0.7 ± 0.4	0.4 ± 0.1	1.3 ± 0.8
*µ*(*rms*)	43 ± 15	57 ± 17	56 ± 21	76 ± 23
*σ*(*rms*)	32 ± 15	41 ± 13	48 ± 28	56 ± 19

**Table 10:** Statistics of time-domain periodicity-based features, in mean and standard deviation.

Feature	Original Signal		Filtered Signal	
	PD	HC	PD	HC
*µ*(*ZC*)	28.1 ± 12.6	36.4 ± 6.8	30.8 ± 13.4	37.4 ± 6.7
*σ*(*ZC*)	36.7 ± 18	47.9 ± 18	44.2 ± 22	49.5 ± 17.1
*µ*(*SSC*)	177.7 ± 41.9	136.4 ± 43.8	174 ± 41.8	138.2 ± 39.9
*σ*(*SSC*)	117.9 ± 24.2	85.1 ± 31.2	118.9 ± 23.3	81.1 ± 30.3

**Table 11:** Statistics of frequency-domain features, in mean and standard deviation.

Feature	Original Signal		Filtered Signal	
	PD	HC	PD	HC
*µ*(*maxf*)	277.5 ± 76.9	457.3 ± 115.9	294.5 ± 94	468.1 ± 199.2
*σ*(*maxf*)	426.7 ± 280.8	690.5 ± 298.5	484.2 ± 321.6	707.4 ± 320.9
*µ*(*waf*)	309.9 ± 85.2	391.7 ± 261.5	327.6 ± 103.3	513.5 ± 155.5
*σ*(*waf*)	401.2 ± 255.5	665.8 ± 271.2	463.4 ± 297.8	697.8 ± 289.1
*µ*(*skw*)	10.8 ± 1	10.9 ± 1	10.9 ± 1	10.3 ± 0.7
*σ*(*skw*)	2.7 ± 0.2	2.9 ± 0.2	2.8 ± 0.2	2.9 ± 0.2
*µ*(*kur*)	136.1 ± 22.5	124.3 ± 12.4	136.7 ± 21	124.3 ± 13.3
*σ*(*kur*)	57.8 ± 3.8	58.9 ± 2.8	58.4 ± 3.7	59.2 ± 2.7

**Table 12:** Statistics of first three formants (*f*_1_, *f*_2_, and *f*_3_), in mean and standard deviation.

Feature	Original Signal		Filtered Signal	
	PD	HC	PD	HC
*µ*(*f*_1_)	122.2 ± 19.4	123.5 ± 12.4	119.9 ± 19	122 ± 11.9
*σ*(*f*_1_)	115.8 ± 13.4	128.2 ± 8.9	116.3 ± 12.9	127.7 ± 8.8
*µ*(*f*_2_)	279.6 ± 57.1	259.5 ± 34.4	274.3 ± 54.2	257 ± 33.4
*σ*(*f*_2_)	218.6 ± 21.6	225.5 ± 7.3	221.7 ± 21.1	226.8 ± 6.4
*µ*(*f*_3_)	787.5 ± 104.5	756.4 ± 61.6	776.5 ± 100.2	751.5 ± 59.4
*σ*(*f*_3_)	383.1 ± 34.5	390.2 ± 16.6	389 ± 29.4	392.4 ± 15.2

**Table 13:** Performance metrics for CNN-based Parkinsonian speech identification.

Feature	Original Signal				Filtered Signal			
	Accuracy	FP	FN	Loss	Accuracy	FP	FN	Loss
Speech spectrograms (all patients)	78%	6	8	0.3	86%	3	5	0.4
Speech spectrograms (reduced dataset)	85%	5	2	0.8	93%	3	0	0.1
Speech energy spectrograms	80%	4	8	0.3	84%	5	5	0.6
Speech energy spectrograms (reduced dataset)	87%	2	4	0.4	96%	2	0	0.1
Mel spectrograms	58%	12	14	0.5	70%	7	10	0.3
Mel spectrograms (reduced dataset)	87%	0	6	0.7	92%	2	2	0.5

**Table 14:** Comparison of the classification accuracy reported in this work vs. the literature, based on the speech task.

Reference	Performance Metrics		
	Speaking Task	Feature	Accuracy
This work	Continuous speech	Speech/speech energy/Mel spectrogram	93%/96%/92%
[41]	n.a.	22 speech attributes	97.4%
[42]	Vowels	19 acoustic features	91.25%/91.23%
[43]	Isolated words	MFCC	60% … 90%
[39]	Sustained vowel a	6 vocal feature sets	89.4%/94.4%
[44]	Sustained phonation, diadochokinetic task, continuous speech	SPEC and MFCC features	>80%
[38]	Short sentence segments	Spectrograms	85.9%
[13]	Sustained vowels	Energy, formants	99.4%
[31]	Continuous speech	Energy	91% … 98%
[28]	Continuous speech	282 features	83% … 93%

n.a.—not available/not reported.

**Table 15:** Comparison of the deep learning-based classification accuracy reported in this work vs. the literature.

Reference	Performance Metrics	
	Classifier	Accuracy
This work	CNN	93%/96%/92%
[44]	CNN	>80%
[38]	CNN	85.9%
[13]	NN	99.4%

## Data Availability

We have chosen not to make the data publicly available in accordance to the protocol statement.

## Appendix A

### Appendix A.1. Wiener Filter Performance Evaluation

**Table A1:** Wiener filter speech enhancement and fidelity measures.

ID	SNR (dB) Original Signal	SNR (dB) Filtered Signal	SNRI	MSE
PD 1	43.1	43.2	0.1	2.29 × 10^−4^
PD 2	46.3	50	3.7	2.27 × 10^−4^
PD 3	44.2	48.3	4.1	7.32 × 10^−5^
PD 4	43.5	43.7	0.2	2.58 × 10^−4^
PD 5	44.9	50.4	5.5	1.5 × 10^−4^
PD 6	42.4	47.8	5.4	3.81 × 10^−4^
PD 7	36.4	42.6	6.2	8.42 × 10^−4^
PD 8	31.8	34.8	3	3.68 × 10^−4^
PD 9	46	49.3	3.3	2.47 × 10^−4^
PD 10	81	81.1	0.1	1.19 × 10^−4^
PD 11	58.2	62.9	4.7	8.94 × 10^−4^
PD 12	44.2	50.9	6.7	2.28 × 10^−4^
PD 13	9.7	11.4	1.7	2.81 × 10^−4^
PD 14	16.1	24.7	8.6	6.5 × 10^−4^
PD 15	26.3	33.3	7	1.94 × 10^−4^
PD 16	15	20.9	5.9	6.93 × 10^−4^
Statistics	39.3 ± 17.4	43.5 ± 16.5	4.1 ± 2.6	2.8 × 10^−4^ ± 2.2 × 10^-4^
HC 1	24	31.3	7.3	3.55 × 10^−4^
HC 2	38.1	44.3	6.2	4.67 × 10^−4^
HC 3	33.7	41.8	8.1	3.47 × 10^−4^
HC 4	27.2	28.6	1.4	3.81 × 10^−4^
HC 5	42.5	46.8	4.3	1.68 × 10^−4^
HC 6	32.2	39	6.8	5.8 × 10^−4^
HC 7	33.5	35.4	1.9	7.49 × 10^−4^
HC 8	44.1	49.5	5.4	3.35 × 10^−4^
HC 9	28.2	32.3	4.1	1.2 × 10^−3^
HC 10	26.3	28.4	2.1	3.79 × 10^−4^
HC 11	51.7	55	3.3	6.67 × 10^−4^
Statistics	34.7 ± 8.6	39.3 ± 8.9	4.6 ± 2.3	5.1 × 10^−4^ ± 2.8 × 10^−4^

### Appendix A.2. Feature Extraction for Parkinsonian Speech Assessment

#### Appendix A.2.1. Phonological Analysis

**Table A2:** Phonological parameters.

ID	Original Signal				Filtered Signal			
	*n_uttering_*	*n_pause_*	*r_speech_*	*t_pause_*	*n_uttering_*	*n_pause_*	*r_speech_*	*t_pause_*
PD 1	13	12	47.4	35	7	6	25.6	1.8
PD 2	19	18	49.7	7.5	14	13	36.6	7
PD 3	17	16	49.1	8.4	14	13	41.1	7.7
PD 4	6	5	35.2	1.5	5	4	29.4	1.4
PD 5	7	6	24.6	4.2	7	6	24.6	4
PD 6	20	19	42.7	10.7	17	16	36.3	10.1
PD 7	13	12	34.1	12.2	13	12	32.1	11.5
PD 8	10	9	34	5.7	9	8	24.9	5.2
PD 9	11	10	43.8	3.5	10	9	39.8	3.2
PD 10	14	13	36.7	7.1	14	13	36.7	5.8
PD 11	7	6	38.2	2.8	7	6	8.2	2.7
PD 12	10	9	32.26	6.6	9	8	29	6.2
PD 13	8	7	28.5	3.6	9	8	32	3.2
PD 14	12	11	33.4	3	14	13	39	5.7
PD 15	19	18	48.2	7.5	18	17	45.6	6.1
PD 16	36	35	52	13.4	34	33	49.1	11.5
Statistics	13.9 ± 7.4	12.9 ± 7.4	39.4 ± 8.3	8.3 ± 7.9	12.6 ± 6.9	11.6 ± 6.9	33.1 ± 9.8	5.8 ± 3.2
HC 1	6	5	19.8	5.2	8	7	24.2	5
HC 2	4	3	16.2	1.3	4	3	16.2	1
HC 3	5	4	21.6	2.1	6	5	25.1	2
HC 4	13	12	34.3	4.4	12	11	34.3	4.2
HC 5	12	11	50	1.9	10	9	35.7	1.7
HC 6	12	11	44.7	5.6	8	7	30.7	5
HC 7	9	8	28	8.9	9	8	28	8.6
HC 8	18	17	51.5	6.2	17	16	49	5.8
HC 9	9	8	29.6	3.4	7	6	23.7	3.3
HC 10	8	7	30.9	3.5	7	6	27.4	3.1
HC 11	7	6	20.8	7.6	7	6	20.8	7.3
Statistics	9.4 ± 4.1	8.4 ± 4.1	31.6 ± 12.3	4.6 ± 2.4	8.6 ± 3.5	7.6 ± 3.5	28.6 ± 8.8	4.3 ± 2.4

#### Appendix A.2.2. Prosody Analysis

**Table A3:** Prosody parameters, in mean and standard deviation.

ID	Original Signal				Filtered Signal			
	*µ*(*I*)	*σ*(*I*)	*µ*(*f*_0_)	*σ*(*f*_0_)	*µ*(*I*)	*σ*(*I*)	*µ*(*f*_0_)	*σ*(*f*_0_)
PD 1	0.075	0.091	113.2	56.6	0.076	0.093	121.12	72.18
PD 2	0.137	0.159	232.8	58.2	0.135	0.157	234.02	57.3
PD 3	0.111	0.122	152.4	36.4	0.111	0.122	155.27	37.83
PD 4	0.105	0.118	138.6	22.5	0.106	0.119	138.62	23.8
PD 5	0.052	0.073	140.3	66.1	0.069	0.097	150.86	69.46
PD 6	0.097	0.122	146.6	29.9	0.097	0.121	147.85	31.11
PD 7	0.143	0.156	127.3	47	0.144	0.157	128.63	44.6
PD 8	0.077	0.093	163.6	78.7	0.081	0.096	161.31	62.25
PD 9	0.119	0.141	120.3	36.4	0.12	0.143	120.98	36.67
PD 10	0.074	0.091	103.1	60.2	0.072	0.09	102.87	59.83
PD 11	0.05	0.07	227.5	57.2	0.07	0.093	232.98	53.89
PD 12	0.02	0.03	136	57.9	0.03	0.041	148.47	69.2
PD 13	0.025	0.035	196.3	82.6	0.033	0.045	206.25	79.66
PD 14	0.04	0.06	135.3	64.2	0.06	0.081	160.64	78.94
PD 15	0.02	0.027	184	105.2	0.024	0.036	190.16	102.28
PD 16	0.02	0.025	202.2	93.5	0.024	0.034	211.98	86.7
Statistics	0.07 ± 0.04	0.09 ± 0.05	157.5 ± 39.8	59.5 ± 22.7	0.07 ± 0.04	0.09 ± 0.04	163 ± 40.4	60.4 ± 21.7
Male statistics	0.08 ± 0.04	0.1 ± 0.04	138.8 ± 33.9	49.8 ± 15.2	0.08 ± 0.03	0.1 ± 0.03	145.3 ± 35.4	54 ± 19
Female statistics	0.07 ± 0.05	0.08 ± 0.06	188.6 ± 18.8	75.8 ± 24.9	0.07 ± 0.05	0.08 ± 0.05	193.2 ± 30.5	71 ± 23.1
HC 1	0.077	0.09	155.04	65.7	0.077	0.088	155.59	63.36
HC 2	0.113	0.113	243.6	37.3	0.144	0.115	245.08	35.01
HC 3	0.102	0.112	235.2	34.5	0.1	0.11	237.23	32.35
HC 4	0.095	0.107	172.6	38.4	0.097	0.111	178.68	46.01
HC 5	0.12	0.134	180.8	47.2	0.12	0.134	180.89	44.81
HC 6	0.075	0.096	128.9	46.1	0.075	0.098	131.12	64.72
HC 7	0.07	0.09	203	98.3	0.076	0.098	203.31	45.85
HC 8	0.08	0.104	156.9	45	0.081	0.106	158.45	99.16
HC 9	0.1	0.1	131.8	50	0.096	0.103	133.56	49.14
HC 10	0.08	0.1	160.1	64.3	0.08	0.099	161.89	65.58
HC 11	0.1	0.121	152	53.9	0.099	0.121	152.1	54.05
Statistics	0.09 ± 0.02	0.1 ± 0.01	174.5 ± 38.2	48.7 ± 23.2	0.09 ± 0.02	0.1 ± 0.01	176.38.2	54.5 ± 18.5
Male statistics	0.08 ± 0.01	0.1 ± 0.01	150.9 ± 17.1	44.1 ± 22.1	0.08 ± 0.01	0.1 ± 0.01	153.2 ± 18.1	64.7 ± 18.9
Female statistics	0.09 ± 0.02	0.01 ± 0.01	202.9 ± 38	54.2 ± 25.8	0.1 ± 0.03	0.1 ± 0.01	203.7 ± 38.8	42.4 ± 8.9

#### Appendix A.2.3. Time-Domain Analysis

**Table A4:** Time-domain intensity-based features, in mean and standard deviation.

ID	Original Signal						Filtered Signal					
	*µ*(*mav*)	*σ*(*mav*)	*µ*(*enrg*)	*σ*(*enrg*)	*µ*(*rms*)	*σ*(*rms*)	*µ*(*mav*)	*σ*(*mav*)	*µ*(*enrg*)	*σ*(*enrg*)	*µ*(*rms*)	*σ*(*rms*)
PD 1	28	17	0.2	0.2	35	21	34	23	0.3	0.3	43	30
PD 2	33	25	0.2	0.3	39	29	43	34	0.4	0.6	50	39
PD 3	22	12	0.1	0.1	26	15	28	17	0.2	0.1	34	20
PD 4	33	23	0.3	0.3	42	29	44	31	0.5	0.5	55	39
PD 5	52	52	0.8	1.2	64	63	70	69	1.4	2.2	86	84
PD 6	35	29	0.3	0.4	41	34	46	59	1.7	4.9	57	116
PD 7	24	14	0.1	0.1	28	16	31	19	0.2	0.2	37	22
PD 8	41	29	0.4	0.5	50	35	53	39	0.7	0.8	65	48
PD 9	29	20	0.2	0.2	36	25	38	27	0.3	0.4	47	34
PD 10	21	13	0.1	0.1	27	17	27	18	0.2	0.2	34	23
PD 11	7	55	1	1.2	79	61	93	75	1.8	2.2	105	83
PD 12	33	22	0.2	0.2	39	25	43	30	0.4	0.4	51	34
PD 13	33	24	0.2	0.4	39	29	40	33	0.4	0.7	48	39
PD 14	50	43	0.6	0.9	61	51	73	60	1.3	1.8	88	71
PD 15	25	23	0.2	0.4	29	26	32	31	0.3	0.6	37	34
PD 16	26	23	0.1	0.4	31	26	32	26	0.2	0.4	38	30
Statistics	36 ± 13	27 ± 13	0.3 ± 0.3	0.5 ± 0.4	43 ± 15	32 ± 15	47 ± 18	38 ± 18	0.7 ± 0.6	0.4 ± 0.1	56 ± 21	48 ± 28
Male statistics	39 ± 15	31 ± 16	0.4 ± 0.3	0.5 ± 0.4	47 ± 17	36 ± 18	52 ± 22	44 ± 22	0.9 ± 0.7	0.6 ± 0.2	63 ± 24	57 ± 32
Female statistics	3 ± 0.7	23 ± 6	0.2 ± 0.1	0.4 ± 0.1	36 ± 0.9	27 ± 7	38 ± 9	30 ± 8	0.4 ± 0.2	0.5 ± 0.3	45 ± 12	35 ± 10
HC 1	39	29	0.3	0.5	47	34	52	39	0.6	0.9	63	0.045
HC 2	49	28	0.5	0.5	59	34	65	39	0.8	0.8	78	0.045
HC 3	42	31	0.4	0.6	49	36	55	43	0.7	1.1	66	0.048
HC 4	41	29	0.4	0.5	49	34	52	39	0.6	0.9	63	0.046
HC 5	26	18	0.2	0.2	32	22	35	25	0.3	0.3	43	0.029
HC 6	45	36	0.5	0.7	58	45	60	48	0.9	1.3	76	0.061
HC 7	62	49	1	1.4	78	64	82	66	1.8	2.6	103	0.086
HC 8	39	30	0.4	0.5	49	38	50	66	0.7	2.6	64	0.086
HC 9	73	47	12	1.2	91	58	98	38	2.1	0.8	121	0.047
HC 10	37	28	0.3	0.4	46	35	48	38	0.6	0.8	61	0.047
HC 11	59	45	0.8	1.1	74	55	78	62	1.5	1.9	98	0.075
Statistics	47 ± 13	34 ± 10	0.5 ± 0.3	0.7 ± 0.4	57 ± 17	41 ± 13	61 ± 18	46 ± 13	1 ± 0.6	1.3 ± 0.8	76 ± 23	56 ± 19
Male statistics	46 ± 14	33 ± 7	0.5 ± 0.3	0.6 ± 0.3	57 ± 17	41 ± 9	60 ± 19	45 ± 11	0.9 ± 0.6	1.2 ± 0.7	75 ± 23	55 ± 16
Female statistics	48 ± 14	34 ± 13	0.6 ± 0.3	0.8 ± 0.5	58 ± 19	42 ± 17	63 ± 19	47 ± 17	1 ± 0.6	1.3 ± 0.9	78 ± 24	57 ± 23

**Table A5:** Time-domain periodicity-based features, in mean and standard deviation.

ID	Original Signal				Filtered Signal			
	*µ*(*ZC*)	*σ*(*ZC*)	*µ*(*SSC*)	*σ*(*SSC*)	*µ*(*ZC*)	*σ*(*ZC*)	*µ*(*SSC*)	*σ*(*SSC*)
PD 1	22.357	16.859	182.544	74.584	22.641	18.677	196.727	82.65
PD 2	29.307	53.375	140.798	140.374	31.872	59.298	140.584	140.668
PD 3	24.325	21.144	121.928	88.092	26.712	29.402	118.478	87.19
PD 4	28.659	29.329	181.151	126.526	29.181	30.003	183.965	128.598
PD 5	66.081	81.9	290.121	173.9	70.194	102.646	279.219	191.997
PD 6	30.005	52.14	204.257	121.608	30.947	54.477	198.394	120.823
PD 7	23.845	40.067	148.127	109.047	24.749	41.65	142.158	107.563
PD 8	34.632	43.872	144.893	104.41	35.116	44.324	150.085	107.657
PD 9	19.442	24.564	180.573	113.788	20.094	25.974	178.236	114.469
PD 10	29.116	39.246	195.915	108.027	29.614	41.167	203.457	110.171
PD 11	21.374	31.819	129.823	123.655	22.56	33.815	132.5	122.207
PD 12	16.494	28.891	174.101	112.079	17.197	31.203	169.326	110.657
PD 13	16.494	33.584	174.101	88.326	30.754	39.367	134.914	93.509
PD 14	37.431	55.588	217.195	128.352	46.013	70.774	223.118	145.956
PD 15	15.75	18.996	169.841	131.924	17.229	23.741	171.263	132.151
PD 16	34.654	18.996	206.441	131.924	37.338	57.353	199.109	116.7
Statistics	28.1 ± 12.6	36.7 ± 18	177.7 ± 41.9	117.9 ± 24.2	30.8 ± 13.4	44.2 ± 22	174 ± 41.8	118.9 ± 23.3
Male statistics	29.5 ± 14.2	40.1 ± 18.9	189.8 ± 43.3	120.4 ± 24.6	31.5 ± 15.8	45.5 ± 25.2	189.3 ± 41.6	122.8 ± 24
Female statistics	25.9 ± 8.5	31.7 ± 14.5	159.7 ± 30	114.2 ± 23.5	29.8 ± 7.1	42.2 ± 14.4	152.4 ± 28.8	113 ± 21.1
HC 1	45.747	76.733	174.158	123.921	47.013	78.947	172.795	125.428
HC 2	38.708	42.538	118.255	76.937	38.446	41.955	117.145	76.903
HC 3	30.326	38.703	154.189	93.201	30.003	40.942	143.291	96.501
HC 4	40.387	62.869	187.823	100.331	41.831	66.345	182.848	109.791
HC 5	32	38.802	90.089	61.733	34.475	43.336	99.189	65.526
HC 6	24.872	27.766	92.931	52.635	26.421	31.123	101.402	57.502
HC 7	39.459	40.397	124.723	55.802	41.166	42.695	132.305	58.475
HC 8	27.968	28.14	84.773	64.632	29.544	42.695	92.793	58.475
HC 9	42.379	58.031	184.803	122.555	42.955	36.452	184.222	54.428
HC 10	35.396	34.743	93.197	50.388	36.469	36.452	96.985	54.428
HC 11	42.988	77.642	195.069	133.854	43.701	78.549	197.261	135.084
Statistics	36.4 ± 6.8	47.9 ± 18	136.4 ± 43.8	85.1 ± 31.2	37.4 ± 6.7	49.5 ± 17.1	138.2 ± 39.9	81.1 ± 30.3
Male statistics	36.1 ± 8.3	48 ± 20.6	136.6 ± 50.7	85.7 ± 34.1	37.4 ± 8	48.7 ± 19.3	138.5 ± 45.7	76.7 ± 32.1
Female statistics	36.7 ± 5.3	47.6 ± 16.9	136.5 ± 39.9	84.3 ± 31.3	37.6 ± 5.4	49.5 ± 16.3	137.8 ± 37.1	86.5 ± 30.7

#### Appendix A.2.4. Frequency-Domain Analysis

**Table A6:** Frequency-domain features in mean value.

ID	Original Signal				Filtered Signal			
	*µ*(*maxf*)	*µ*(*waf*)	*µ*(*skw*)	*µ*(*kur*)	*µ*(*maxf*)	*µ*(*waf*)	*µ*(*skw*)	*µ*(*kur*)
PD 1	209.8221	224.8454	9.976358	117.5921	190.2661	209.5559	10.33465	125.1718
PD 2	360.1689	416.25	11.92247	159.6	414.8776	444.9981	11.83087	157.4186
PD 3	370.1667	366.0654	9.967189	117.7195	375.2765	383.3179	9.904568	116.8925
PD 4	250.2299	306.0685	10.05182	119.875	267.008	305.6195	10.13883	121.6912
PD 5	302.4605	370.1943	9.733266	113.4279	300.2343	374.7697	10.01356	118.778
PD 6	230.2874	260.1802	12.05196	161.9582	230.490524	268.8687	12.06773	162.2545
PD 7	220.8475	253.263	10.69389	130.4106	223.762915	250.1917	10.76735	132.0026
PD 8	345.9367	411.2698	10.22188	123.6549	355.175689	417.4254	10.17484	122.818
PD 9	182.4607	208.4106	10.10069	119.5384	184.348562	210.2118	10.139	120.2532
PD 10	305.2498	319.7704	9.385109	106.0929	296.426479	327.8041	9.545114	109.7087
PD 11	249.8765	277.7127	13.11219	185.5506	262.681159	289.9209	13.00803	183.6191
PD 12	146.2178	154.7583	11.97501	158.6683	144.605475	157.4248	11.97299	158.6024
PD 13	315.6509	354.7091	10.87591	136.1331	365.054945	395.4144	10.86759	136.3212
PD 14	298.2712	336.0759	10.39996	125.5157	430.044276	463.8346	10.22208	122.7967
PD 15	219.5424	235.0433	11.66939	152.9737	225.228311	233.9798	11.6668	153.0658
PD 16	433.1558	464.4558	11.40796	149.6136	447.3	508.7318	11.26647	146.6707
Statistics	277.5 ± 76.9	309.9 ± 85.2	10.8 ± 1	136.1 ± 22.5	294.5 ± 94	327.6 ± 103.3	10.9 ± 1	136.7 ± 21
Male statistics	239.6 ± 53.9	271.1 ± 63.1	10.7 ± 1.2	133.9 ± 25.9	253 ± 77	285.8 ± 85.4	10.8 ± 1.1	135.5 ± 24.1
Female statistics	340.8 ± 70.9	374.6 ± 78.9	11 ± 0.8	139.9 ± 16.9	363.8 ± 76.1	397.3 ± 91.6	11 ± 0.8	138.9 ± 16.5
HC 1	623.3607	657.5876	9.915721	118.046	647.812359	682.7459	9.895411	117.3672
HC 2	477.2542	522.7215	11.13494	142.5209	451.206897	516.0038	11.10386	141.5863
HC 3	301.9284	349.3909	11.5549	149.9868	288.343558	345.2166	11.67933	152.7823
HC 4	343.8331	397.1155	10.73022	132.7825	370.254314	428.8957	10.85112	135.3927
HC 5	473.8431	510.7556	10.06492	121.1023	506.415344	553.0855	9.929213	118.6239
HC 6	340.6038	380.7002	9.451434	107.9514	358.076225	400.7224	9.440863	107.6968
HC 7	641.3078	678.6393	10.06799	120.9231	642.514345	691.8391	10.03913	120.6899
HC 8	448.7052	474.8081	10.08178	119.5018	475.053763	496.7997	10.04414	119.2715
HC 9	367.6768	403.4554	9.790699	114.6094	376.106195	410.718	9.844548	115.3877
HC 10	571.8169	627.1641	9.956054	118.8349	588.329839	623.486	9.8651	116.949
HC 11	439.9353	501.9098	10.13185	121.2875	444.933078	498.9354	10.14959	121.573
Statistics	457.3 ± 115.9	500.4 ± 114.5	10.9 ± 1	124.3 ± 12.4	468.1 ± 199.2	513.5 ± 155.5	10.3 ± 0.7	124.3 ± 13.3
Male statistics	449.3 ± 122.4	490.1 ± 122.6	10.9 ± 1.3	118.6 ± 8.1	469.3 ± 124	507.3 ± 199.4	10 ± 0.5	118.7 ± 9.1
Female statistics	466.9 ± 121	512.7 ± 116.6	11 ± 0.8	131.2 ± 14	466.7 ± 127.5	521 ± 124.1	10.6 ± 0.8	131 ± 15.3

**Table A7:** Frequency-domain features, in standard deviation.

ID	Original Signal				Filtered Signal			
	*σ*(*maxf*)	*σ*(*waf*)	*σ*(*skw*)	*σ*(*kur*)	*σ*(*maxf*)	*σ*(*waf*)	*σ*(*skw*)	*σ*(*kur*)
PD 1	140.8132	110.2558	2.633903	55.30804	123.2352	109.5277	2.743987	58.12588
PD 2	785.5251	789.5384	2.994304	63.92157	989.0715	887.1457	2.978835	63.58607
PD 3	258.7677	197.0151	2.825177	57.78512	298.5884	288.7914	2.904553	58.90765
PD 4	286.0091	282.4953	2.691118	55.13494	317.0508	263.9887	2.731828	55.89014
PD 5	528.8487	544.7161	2.818697	56.79514	536.2376	547.2557	2.781482	56.80749
PD 6	607.7036	544.687	2.760685	60.40223	623.1119	592.8185	2.748375	60.312
PD 7	442.4261	451.3987	2.440274	53.07628	459.0798	459.009	2.424676	52.861
PD 8	437.354	463.7074	2.910983	59.8237	456.3991	482.6861	2.93604	59.88946
PD 9	89.73631	70.23631	2.505477	52.43359	88.24194	77.2381	2.538476	53.54082
PD 10	584.4737	458.1556	2.729204	56.43168	588.0268	518.4518	2.80797	58.18234
PD 11	253.676	264.5755	2.713397	58.55941	305.6971	313.7641	2.803873	60.36714
PD 12	62.34063	66.32817	2.503306	56.13981	60.54761	98.17067	2.463561	55.52048
PD 13	317.5697	296.6603	2.728088	58.53734	544.5609	494.1021	2.76784	58.32652
PD 14	781.5125	698.909	2.621361	54.91706	1089.867	990.5238	2.743447	56.2561
PD 15	199.1553	255.0108	2.662286	58.29154	255.5855	279.8706	2.659547	58.0698
PD 16	1051.378	926.1861	3.181141	67.42014	1012.541	1020.364	3.235481	68.03222
Statistics	426.7 ± 280.8	401.2 ± 255.5	2.7 ± 0.2	57.8 ± 3.8	484.2 ± 321.6	4634 ± 297.8	2.8 ± 0.2	58.4 ± 3.7
Male statistics	377.8 ± 260.3	349.2 ± 236.3	2.6 ± 0.8	55.9 ± 17	419.1 ± 323.1	380.4 ± 304.7	2.7 ± 0.9	56.8 ± 18.1
Female statistics	508.3 ± 338	488 ± 303	2.9 ± 0.2	61 ± 3.8	592.8 ± 333	575.5 ± 309.8	2.9 ± 0.2	61.1 ± 3.9
HC 1	1410.013	1295.124	3.047597	60.42438	1434.005	1370.838	3.010594	60.23597
HC 2	600.6572	546.8154	2.948929	62.02396	508.5495	533.5049	2.916	61.50816
HC 3	317.4417	359.8861	2.664837	58.45661	252.831	320.5499	2.647889	57.81285
HC 4	709.2429	659.4237	2.741509	56.89116	725.7896	736.6574	2.765028	57.35001
HC 5	741.7255	707.3263	3.074998	61.17489	841.5044	806.4467	3.18064	62.78911
HC 6	476.0353	457.6013	2.816181	56.1085	531.2828	506.766	2.814497	55.93618
HC 7	915.6143	873.083	3.062648	61.8054	964.6048	903.3665	3.124569	62.63882
HC 8	433.6057	445.4268	2.558988	52.90653	538.7827	523.2871	2.677256	54.6878
HC 9	469.309	410.9073	2.850247	59.04572	398.2593	419.0779	2.801386	58.13629
HC 10	731.1745	751.7804	3.00003	60.84957	764.2004	758.5625	3.039602	61.60967
HC 11	790.7127	816.42	2.873028	57.9772	821.1247	796.3562	2.871022	58.41921
Statistics	690.5 ± 298.5	665.8 ± 271.2	2.9 ± 0.2	58.9 ± 2.8	707.4 ± 320.9	697.8 ± 289.1	59.2 ± 0.2	59.2 ± 2.7
Male statistics	704.9 ± 368.6	670 ± 334.7	2.8 ± 0.2	57.7 ± 3	732 ± 369.6	719.2 ± 346.4	2.9 ± 0.14	58 ± 2.6
Female statistics	673.2 ± 288.6	660.7 ± 209.2	2.9 ± 0.2	60.3 ± 1.9	677.7 ± 291	672 ± 239.7	2.9 ± 0.2	60.6 ± 2.4

##### Appendix A.2.5. LPC Analysis

**Table A8:** First three formants (*f*_1_, *f*_2_, and *f*_3_), in mean value.

ID	Original Signal			Filtered Signal		
	*µ*(*f*_1_)	*µ*(*f*_2_)	*µ*(*f*_3_)	*µ*(*f*_1_)	*µ*(*f*_2_)	*µ*(*f*_3_)
PD 1	146.3977	356.2246	927.9631	95.99813	215.8823	664.4091
PD 2	140.5878	305.1284	846.6479	143.1003	309.3127	853.4781
PD 3	116.3885	238.901	723.2804	117.1406	241.6413	728.9491
PD 4	116.3885	238.901	723.2804	112.4424	251.6562	725.0138
PD 5	106.2606	244.0261	718.9596	107.1858	246.2775	720.2278
PD 6	117.6647	295.6761	806.0591	128.2465	321.9942	865.6273
PD 7	126.7025	318.2057	858.2249	124.9557	246.3748	721.9826
PD 8	125.9183	249.031	725.5743	118.7285	290.2411	808.252
PD 9	118.2062	286.9403	799.7578	92.24254	199.7526	636.9858
PD 10	90.98081	195.1003	631.759	167.9252	397.4367	1007.766
PD 11	168.3419	398.1365	1008.629	128.3763	346.5467	900.6316
PD 12	127.7762	344.6129	898.1218	112.3823	232.116	714.1573
PD 13	110.4784	225.7373	701.0682	106.2781	235.6601	703.9548
PD 14	102.7345	230.3552	686.8945	139.465	323.6074	864.4784
PD 15	138.2548	319.142	857.9386	105.7259	234.3006	699.3675
PD 16	101.5309	227.0754	685.9352	128.2465	321.9942	865.6273
Statistics	122.2 ± 19.4	279.6 ± 57.1	787.5 ± 104.5	119.9 ± 19	274.3 ± 54.2	776.5 ± 100.2
Male statistics	122.1 ± 23.1	290.8 ± 67.5	806 ± 124	117.6 ± 21.7	280.2 ± 63.8	784.1 ± 118.8
Female statistics	122.2 ± 15.5	260.8 ± 40.9	756.7 ± 75.6	123.8 ± 15	264.6 ± 40.8	763.7 ± 74.5
HC 1	119.4577	258.2862	747.4789	119.796	259.1702	748.1364
HC 2	126.8611	243.3643	731.9308	125.3441	241.6419	726.2462
HC 3	127.005	279.1861	794.3075	127.6849	281.9603	800.3555
HC 4	118.1437	270.415	773.6646	120.3063	276.3826	780.0706
HC 5	141.4042	302.962	837.551	137.2403	293.8991	823.2496
HC 6	139.2445	317.8754	863.2708	135.1576	308.9066	846.237
HC 7	112.6445	209.2341	670.1639	109.3551	204.5748	661.7582
HC 8	137.7004	275.2643	781.1636	135.5184	269.6477	769.8928
HC 9	100.6592	212.7324	669.9564	98.78291	210.2192	667.7949
HC 10	116.4745	231.9436	720.1556	115.1922	230.5635	717.091
HC 11	119.2252	253.3964	731.0832	117.1745	249.7392	725.2645
Statistics	123.5 ± 12.4	259.5 ± 34.4	756.4 ± 61.6	122 ± 11.9	257 ± 33.4	751.5 ± 59.4
Male statistics	121.9 ± 14.5	261 ± 36.6	759.3 ± 65	120.8 ± 13.7	25,901 ± 34.9	754.9 ± 60.4
Female statistics	125.5 ± 10.7	257.6 ± 35.6	753 ± 64.5	123.4 ± 10.6	254.4 ± 35.3	747.4 ± 64.9

**Table A9:** First three formants (*f*_1_, *f*_2_, and *f*_3_), in standard deviation.

ID	Original Signal			Filtered Signal		
	*σ*(*f*_1_)	*σ*(*f*_2_)	*σ*(*f*_3_)	*σ*(*f*_1_)	*σ*(*f*_2_)	*σ*(*f*_3_)
PD 1	105.5148	191.726	310.0546	119.8245	233.1591	400.3893
PD 2	119.8571	217.726	389.5605	120.1219	215.9192	385.9008
PD 3	130.1879	230.3062	397.0764	129.7914	230.2576	396.8608
PD 4	122.6321	231.428	394.7242	122.7717	232.5018	395.1004
PD 5	116.6938	228.0452	406.5741	117.4288	229.7301	407.7685
PD 6	104.8515	225.3515	407.9145	105.2166	224.507	406.5868
PD 7	104.7348	213.5573	383.3348	105.0007	212.0539	379.9155
PD 8	132.904	230.5034	400.8092	133.1788	230.791	402.9661
PD 9	109.764	223.279	404.8753	108.5854	222.2566	401.8178
PD 10	125.4507	234.265	382.4851	125.3875	236.2768	384.0149
PD 11	91.01827	153.9159	291.0029	90.96241	155.512	290.7811
PD 12	93.42048	202.0746	365.9657	92.79807	200.2818	362.8614
PD 13	131.167	230.5023	395.655	130.7768	231.1815	396.792
PD 14	131.167	230.5023	395.655	123.4698	240.062	412.6428
PD 15	111.0153	210.9409	389.2035	110.2592	209.8486	386.3314
PD 16	122.5947	242.9469	415.3833	124.49	242.7555	413.6597
Statistics	115.8 ± 13.4	218.6 ± 21.6	383.1 ± 34.5	116.3 ± 12.9	221.7 ± 21.1	389 ± 29.4
Male statistics	110.5 ± 35.6	213.4 ± 68.6	374.3 ± 119.4	111.1 ± 35.6	218.6 ± 70.1	384.2 ± 120.8
Female statistics	124.6 ± 8.4	227.2 ± 11.3	397.9 ± 9.6	124.8 ± 8.5	226.8 ± 11.9	397 ± 10.5
HC 1	125.2216	234.0009	408.6084	125.0871	234.0544	408.5006
HC 2	141.3759	229.5698	376.7384	140.8267	230.6014	378.0453
HC 3	121.4583	222.5808	394.6395	120.9949	222.8789	393.4422
HC 4	121.4583	222.5808	394.6395	120.9949	222.8789	393.4422
HC 5	123.4126	216.2284	379.077	123.3718	219.2568	386.5237
HC 6	116.3071	210.4272	368.4274	116.2192	214.4403	379.4195
HC 7	144.1913	230.2897	362.6656	143.1769	232.56	363.4399
HC 8	131.9069	223.022	397.9898	132.8698	225.197	403.2771
HC 9	125.6425	230.4328	402.9594	124.1504	230.823	402.7296
HC 10	135.9895	232.2704	391.454	134.6502	232.8903	391.5255
HC 11	123.2632	229.5243	415.2614	122.8076	229.6399	416.1476
Statistics	128.2 ± 8.9	225.5 ± 7.3	390.2 ± 16.6	127.7 ± 8.8	226.8 ± 6.4	392.4 ± 15.2
Male statistics	126.1 ± 7.1	225.5 ± 8.8	394 ± 13.9	125.7 ± 7	226.7 ± 7.4	396.5 ± 10.5
Female statistics	130.7 ± 11.1	225.6 ± 6.1	385.7 ± 20.1	130.2 ± 10.8	227 ± 5.7	387.5 ± 19.5
